# Capitalizing on genebank core collections for rare and novel disease resistance loci to enhance barley resilience

**DOI:** 10.1093/jxb/erae283

**Published:** 2024-06-27

**Authors:** Zhihui Yuan, Maximilian Rembe, Martin Mascher, Nils Stein, Murukarthick Jayakodi, Andreas Börner, Klaus Oldach, Ahmed Jahoor, Jens Due Jensen, Julia Rudloff, Viktoria-Elisabeth Dohrendorf, Luisa Pauline Kuhfus, Emmanuelle Dyrszka, Matthieu Conte, Frederik Hinz, Salim Trouchaud, Jochen C Reif, Samira El Hanafi

**Affiliations:** Leibniz Institute of Plant Genetics and Crop Plant Research (IPK) Gatersleben, Seeland, Germany; Leibniz Institute of Plant Genetics and Crop Plant Research (IPK) Gatersleben, Seeland, Germany; KWS SAAT SE & Co. KGaA, Grimsehlstr. 31, D-37574 Einbeck, Germany; Leibniz Institute of Plant Genetics and Crop Plant Research (IPK) Gatersleben, Seeland, Germany; German Centre for Integrative Biodiversity Research (iDiv) Halle-Jena-Leipzig, Leipzig, Germany; Leibniz Institute of Plant Genetics and Crop Plant Research (IPK) Gatersleben, Seeland, Germany; Crop Plant Genetics, Institute of Agricultural and Nutritional Sciences, Martin-Luther-University of Halle-Wittenberg, Halle (Saale), Germany; Leibniz Institute of Plant Genetics and Crop Plant Research (IPK) Gatersleben, Seeland, Germany; Leibniz Institute of Plant Genetics and Crop Plant Research (IPK) Gatersleben, Seeland, Germany; KWS LOCHOW GmbH, Ferdinand-von-Lochow-Str. 5, D-29303 Bergen, Germany; Nordic Seed Germany GmbH, Kirchhorster Str. 16, D-31688 Nienstädt, Germany; Nordic Seed Germany GmbH, Kirchhorster Str. 16, D-31688 Nienstädt, Germany; Limagrain GmbH, Salderstr. 4, D-31226 Peine-Rosenthal, Germany; Nordsaat Saatzucht GmbH, Zuchtstation Gudow, Hofweg 8, D-23899 Gudow, Germany; Syngenta France SAS, 12 Chemin de l’hobit, BP 27, 31790, Saint-Sauveur, France; Syngenta France SAS, 12 Chemin de l’hobit, BP 27, 31790, Saint-Sauveur, France; Syngenta France SAS, 12 Chemin de l’hobit, BP 27, 31790, Saint-Sauveur, France; SAATZUCHT BAUER GmbH & CO.KG, Landshuter Straße 3a, D-93083 Obertraubling, Germany; Secobra Saatzucht GmbH, Feldkirchen 3, D-85368 Moosburg an der Isar, Germany; Leibniz Institute of Plant Genetics and Crop Plant Research (IPK) Gatersleben, Seeland, Germany; Leibniz Institute of Plant Genetics and Crop Plant Research (IPK) Gatersleben, Seeland, Germany; CIMMYT, Mexico

**Keywords:** Barley, core collection, disease resistance, genome-wide association analysis, plant genetic resources, rare and novel alleles

## Abstract

In the realm of agricultural sustainability, the utilization of plant genetic resources for enhanced disease resistance is paramount. Preservation efforts in genebanks are justified by their potential contributions to future crop improvement. To capitalize on the potential of plant genetic resources, we focused on a barley core collection from the German *ex situ* genebank and contrasted it with a European elite collection. The phenotypic assessment included 812 plant genetic resources and 298 elites, with a particular emphasis on four disease traits (*Puccinia hordei*, *Blumeria graminis hordei*, *Ramularia collo-cygni*, and *Rhynchosporium commune*). An integrated genome-wide association study, employing both Bayesian-information and linkage-disequilibrium iteratively nested keyway (BLINK) and a linear mixed model, was performed to unravel the genetic underpinnings of disease resistance. A total of 932 marker–trait associations were identified and assigned to 49 quantitative trait loci. The accumulation of novel and rare resistance alleles significantly bolstered the overall resistance level in plant genetic resources. Three plant genetic resources donors with high counts of novel/rare alleles and exhibiting exceptional resistance to leaf rust and powdery mildew were identified, offering promise for targeted pre-breeding goals and enhanced resilience in future varieties. Our findings underscore the critical contribution of plant genetic resources to strengthening crop resilience and advancing sustainable agricultural practices.

## Teaser

Novel and rare resistance allele accumulation empowered the selection of promising plant genetic resources, offering potential for targeted pre-breeding goals and enhanced resilience in future varieties

## Introduction

Barley (*Hordeum vulgare* L.) is one of the oldest domesticated crops, and ranks fourth in cereal production behind wheat (Horsley and Hochhalter, 2004). Its versatility extends beyond its traditional uses as malting and feed, encompassing diverse food and beverage applications (Langridge, 2018). Despite its prominence, barley production encounters diverse challenges, primarily due to the persistent threat of diseases. Significant yield and economic losses have been reported worldwide (Havis *et al.*, 2015; Ababa *et al.*, 2023; Dracatos *et al.*, 2023), due to many different diseases, such as leaf rust (caused by *Puccinia hordei*), powdery mildew (caused by *Blumeria graminis hordei*), leaf spots (caused by *Ramularia collo-cygni*), and scald (caused by *Rhynchosporium commune*), necessitating innovative strategies for sustainable control. The landscape of barley diseases is dynamic, marked by the breakdown of host resistance, evolving pathogen strains, and the emergence of threats once deemed to be minor. Fungicide insensitivity further compounds the complexity of disease management, underscoring the urgent requirement for effective and sustainable approaches. In this context, resistance breeding, an economic and environment-friendly approach, is critical for sustainably controlling these pathogens (Mundt, 2014). Plant disease resistance can be classified into qualitative resistance and quantitative resistance: the qualitative resistance determined by single loci [genes and/or quantitative trait loci (QTLs)] can often be easily overcome by new strains of a pathogen, whereas resistance conferred by the accumulation of quantitative resistance genes with a complex genetic architecture, although not absolute, tends to be more durable over time (Miedaner and Korzun, 2012; Walters *et al.*, 2012; Camenzind *et al.*, 2024).

In the past few decades, many major resistance genes and QTLs have been identified and deployed against barley pathogens (Havis *et al.*, 2015; Park *et al.*, 2015; Kusch and Panstruga, 2017; Zhang *et al.*, 2020; Gou *et al.*, 2023). Nevertheless, the ever-changing nature of pathogens requires continuous adaptation. Despite this challenge, modern breeding programs rely largely on recycling elite materials (Nelson *et al.*, 2018), leading to a relatively narrowed genetic diversity and strong selection at the resistance loci, thereby limiting the potential for discovering useful novel genes (Chełkowski *et al.*, 2003). Expanding beyond these constraints lies the promise of delving into the vast genetic reservoirs present within plant genetic resources (PGRs; Khoury *et al.*, 2010). This untapped potential holds great significance for biodiversity assessment and maintenance efforts. A strategic core collection approach becomes instrumental in maximizing genetic diversity and unearthing novel sources of resistance that can navigate the challenges posed by evolving pathogen strains.

Our study addresses these challenges by focusing on a spring and winter barley core collection, including 812 PGRs selected from the Federal *ex situ* Genebank for Agricultural and Horticultural Crop Plants maintained at IPK in Gatersleben, Germany, and 298 European elite varieties. The entire population was phenotyped in multienvironmental trials for three agronomically important traits: plant height (PH), heading date (HD), and lodging (LOD), and four disease traits: *Puccinia hordei* (PUC), *Blumeria graminis hordei* (BLU), *Ramularia collo-cygni* (RAM), and *Rhynchosporium commune* (RHY), with the aim to harness valuable genes and alleles as a means to enhance disease breeding programs. In particular, our objectives were to (i) evaluate the phenotypic variability of 1110 diverse barley genotypes for the aforementioned traits, (ii) detect loci associated with resistance against the disease traits, (iii) identify rare and novel candidate resistance alleles with low frequencies in the elite group, and (iv) select the most promising resistant PGR donors that maximize the number of rare and novel candidate resistance alleles that can be further utilized in future pre-breeding programs.

## Materials and methods

### Plant material and phenotypic data analysis

**Fig. 1.**
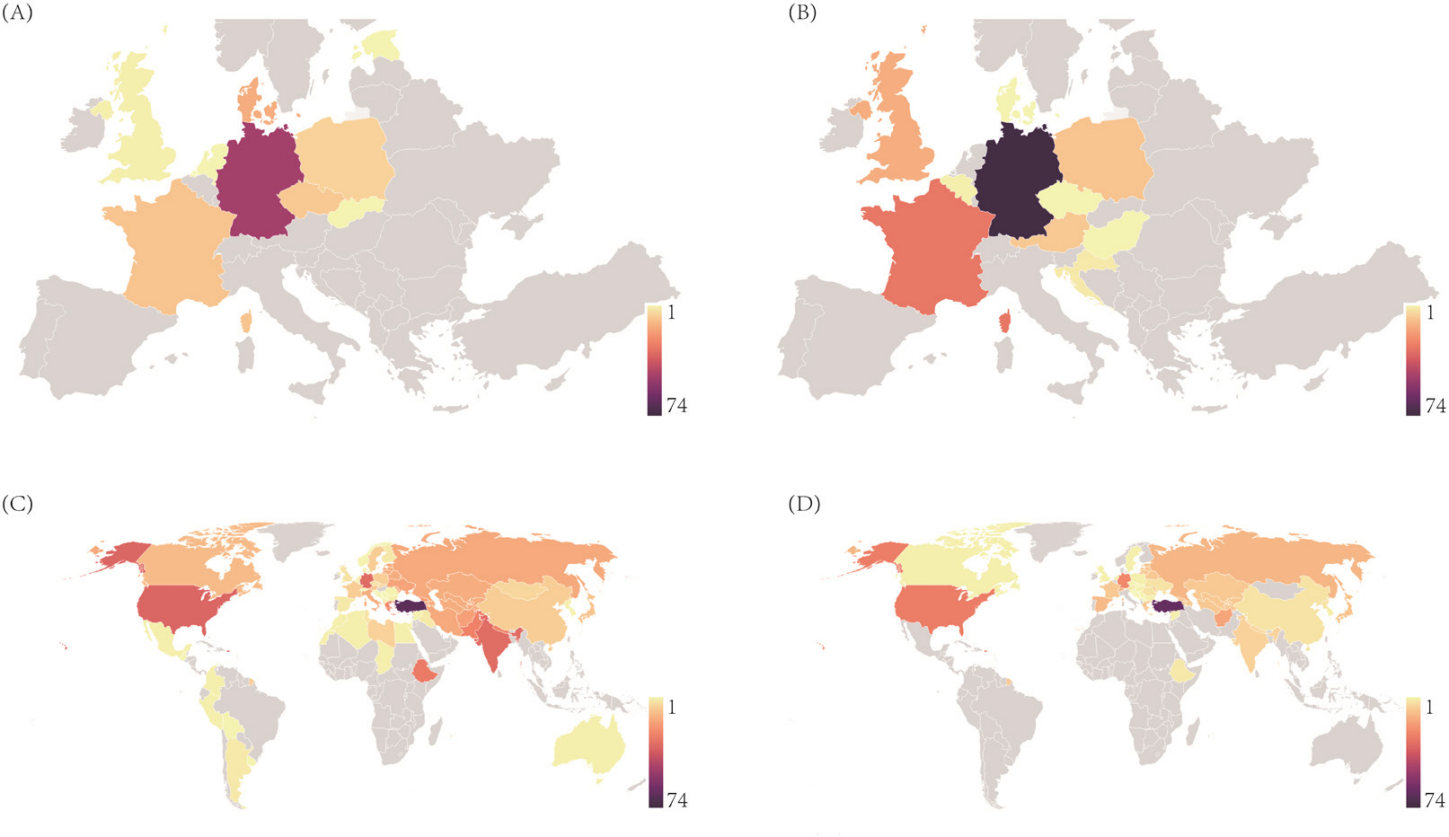
Geographical distribution of the collection of 1110 barley genotypes. Geographical distribution of elite lines in spring type (A), elite lines in winter type (B), plant genetic resources in spring type (C), and plant genetic resources in winter type (D). The color of a country reflects the number of genotypes originating from there.

This study utilized a total of 1110 genotypes that covered diverse geographic origins (Fig. 1; Supplementary Table S1). The collection was systematically categorized into four subgroups: elites in winter (Elite_Winter, *n*=170) and spring type (Elite_Spring, *n*=128), together with PGRs in winter (PGR_Winter, n=288) and spring type (PGR_Spring, *n*=524). With the aim to capture a good representation of the barley diversity space, the PGRs were thoughtfully selected from a previously described barley core 1000 collection (Oppermann *et al.*, 2015; Milner *et al.*, 2019), based on their performance during seed regeneration. The 812 PGR pool originated from 57 countries spanning five continents, while the remaining 298 elite lines were exclusively from Europe. The field trials were conducted in eight environments throughout Germany in three consecutive years: 2020, 2021, and 2022, following a generalized alpha lattice design and considering two-row plots (1 m^2^) as the experimental unit.

The phenotypic data encompass three agronomy traits (HD: measured in days from 1 January for winter types and from the sowing date onward for the spring types, PH, and LOD), and four disease traits (PUC, BLU, RAM, and RHY) evaluated under natural infection. The disease severities were scored using an ordinal scale from 1 (fully resistant) to 9 (fully susceptible) referring to the German Federal Plant Variety Office guideline (Bundessortenamt, 2000).

Statistical analysis was separately conducted for spring and winter barley phenotypic data. An initial step involved removal of outliers using Tukey’s method (Anscombe and Tukey, 1963). Subsequently, a linear mixed model (LMM) was employed to analyze the generated data across environments:


$$\begin{array}{l} y_{ijkm}=\mu +E_{m}+g_{i}+g_{i}\times E_{m}+E_{m}:r_{j}:b_{k}+e_{ijkm} \end{array}$$ (1)

where *y*_ijkm_ is the vector of phenotypic records for the ith genotype (*g*) tested in the kth bock (*b*) nested in the jth replication (*r*) in the mth environment (*E*), μ was the common mean, and *e* denoted the error term of the model. We assumed that all random effects followed an independent normal distribution with different variance components. In model (1), all terms except µ and *g*_i_ were treated as random to compute the best linear unbiased estimations (BLUEs) across environments, whereas all terms except µ were modeled as random to estimate variance component and broad-sense heritability as:


$$\begin{array}{l} \overset{2}{h}=\frac{\sigma_{g}^{2}}{\sigma_{g}^{2}+\frac{\sigma_{g\times E}^{2}}{\bar{n_{E}}}+\frac{\sigma_{e}^{2}}{\bar{n_{R}}}}, \end{array}$$ (2)

where $\sigma_{g}^{2}$, $\sigma_{g\times E}^{2}$, and $\sigma_{e}^{2}$ are the estimates of the corresponding genotypic, interaction of genotype and environment, and residual variances, respectively. $\bar{n_{R}}$ is the number of replications per genotype, and $\bar{n_{E}}$ is the average number of environments in which the genotypes were phenotyped.

All LMM equations were fitted using the ASReml-R package (version 4; Butler *et al.*, 2023). The estimated BLUEs were then used to conduct a genome-wide association study (GWAS). Pearson’s correlation coefficients between traits were further analyzed using the R package corrplot (version 0.92; Wei and Simko, 2017). In order to investigate the relationships between the traits, path analysis incorporating a multiple linear regression model (MLRM) was conducted using the R package lavaan (version 0.6-16; Rosseel, 2012), and visualized using the R package semPlot (version 1.1.6; Epskamp, 2015).

### Genotypic data and diversity analysis

Whole-genome sequencing (WGS) data of the 1110 genotypes were generated at IPK Gatersleben using the Illumina NovaSeq 6000 platform (Jayakodi *et al.*, 2024, Preprint). Raw sequencing reads were trimmed using cutadapt (version 3.3; Martin, 2011) and aligned to the MorexV3 reference genome (Mascher *et al.*, 2021) using Minimap2 (version 2.20; Li, 2018). The resultant alignment records were sorted with Novosort (V3.09.01; http://www.novocraft.com). On average, the coverage was 4.7× across all samples, with a minimum of 0.5× and a maximum of 22.6×. A total of 149 380 812 single nucleotide polymorphisms (SNPs) were initially outputted by BCFtools (version 1.9; Li, 2011).

Genome-wide linkage disequilibrium (LD) analysis of the four subgroups was carried out separately by determining the pairwise squared allele frequency correlations (*r*^2^) with previously described formulas reported by Hill and Robertson (1968). LD within a specific genomic region (2 Mb) was calculated and visualized by PopLDdecay (version 3.40; Zhang *et al.*, 2019). In order to validate the population structure of spring and winter barley accessions, principal coordinate analysis (PCoA) was conducted using the R package ape (v5.7-1; Paradis and Schliep, 2019) based on pairwise Rogers’ distance among genotypes.

Afterward, SNP markers were individually filtered with the index of minor allele frequency ≥0.05 and missing rate <0.05 on the basis of the four subgroups. After this filtering, the remaining missing values were phased and imputed by Beagle (version 5.2; Browning *et al.*, 2018). Subsequently, an *r*^2^ cut-off of 0.2 was set to prune markers PLINK (version 1.9; Purcell *et al.*, 2007) with a sliding window size of 50 kb, and a step size of 10 kb. The final number of SNPs available for GWAS differed in the four subgroups due to the aforementioned quality control process: 710 855 of Elite_Spring, 945 074 of Elite_Winter, 2 321 327 of PGR_Spring, and 1 775 972 of PGR_Winter. For each tested SNP, homozygous for the most frequent allele, heterozygous, and homozygous for the alternative allele were coded as 0, 1, and 2, respectively. The correlation between Rogers’ distance and the absolute trait differences (Euclidean distance) in each subgroup was tested through a Mantel test implemented in the vegan R package (v2.6-4; Oksanen *et al.*, 2022).

### Genome-wide association analysis by mixed models

A GWAS was conducted based on the integration of genotypic information of the four subgroups with their corresponding phenotypic data. In order to avoid possible overcorrection of associations, BLINK (Huang *et al.*, 2019) and LMM were used to identify significant SNPs for the seven traits. The two models were implemented using the statistical genetics R packages Genome Association and Prediction Integrated Tool (GAPIT; Lipka *et al.*, 2012) and Genome-wide Efficient Mixed-Model Association (GEMMA; Zhou and Stephens, 2012), respectively. The top three eigenvectors in a principal component analysis (PCA) and the kinship matrix were computed using the pipelines embedded in GAPIT and GEMMA, respectively, to control the population structure for GWAS, and hence reduce the inflation of false-positive associations.

Significant association thresholds were defined using the Bonferroni correction (Holm, 1979; 0.05/the number of SNPs) for each group to control false positives. Subsequently, Manhattan and combined quantile–quantile plots were generated using the R package CMplot (version 4.3.1; Yin *et al.*, 2021). For calculating the proportion of variance in phenotype explained by a given SNP (PVE), sample size, minor allele frequency, effect size, and the standard error of each SNP were fitted into a linear regression model previously described by Shim *et al.* (2015).

### QTL identification by the extent of linkage disequilibrium-based blocks

Since LD is extremely variable among different loci and the breakdown of LD is often discontinuous and presents a haploblock-like structure, the usage of the average *r*^2^ on the whole genome basis may distort the QTL identification of each locus (Daly *et al.*, 2001). Therefore, the SNPs were clustered based on LD using the function of haplotype block estimation ‘--clump’ in PLINK (version 1.9), shifting the identification of a block from a given average distance on a chromosome to grouping significant SNPs that are inherited together into different blocks due to their tight LD. The function identifies independent blocks which are defined as a proximal set of SNPs with minimum pairwise LD that are larger than a pre-defined threshold (*r*^2^=0.2). A maximum distance threshold between SNP pairs was set as 10 Mb to avoid unrealistically large blocks. Markers that could not be assigned to any block were treated as individual blocks with just one SNP. The QTL was then defined as the significant markers’ corresponding blocks. The physical positions of all QTLs spanned by the haplotype block were determined by aligning their reference sequences to a barley reference genome MorexV3 (Mascher *et al.*, 2021) in order to explore annotated genes inside or overlapping with each QTL.

### Resistance allele identification

In our study, the resistance allele denoted the allele that conferred enhanced resistance to each disease. More precisely, the effect of each significant marker was determined based on the output marker effects. If the marker effect is negative, the minor allele is deemed to decrease the disease severity, and is thus defined as the resistance allele. Conversely, if the marker effect is positive, the major allele was defined as the resistance allele. Afterward, all the significant markers were categorized for each growth habit to calculate their resistance allele frequency (RAF) within the elite and PGR pools separately. The aim was to filter out novel resistance alleles (NRAs) where the RAF in the elite pool (RAF_Elite_)=0, and rare resistance alleles (RRAs) where 0<RAF_Elite_<0.05.

## Results

### Analyses of the barley core collection revealed broad genetic diversity and apparent population structure

**Fig. 2.**
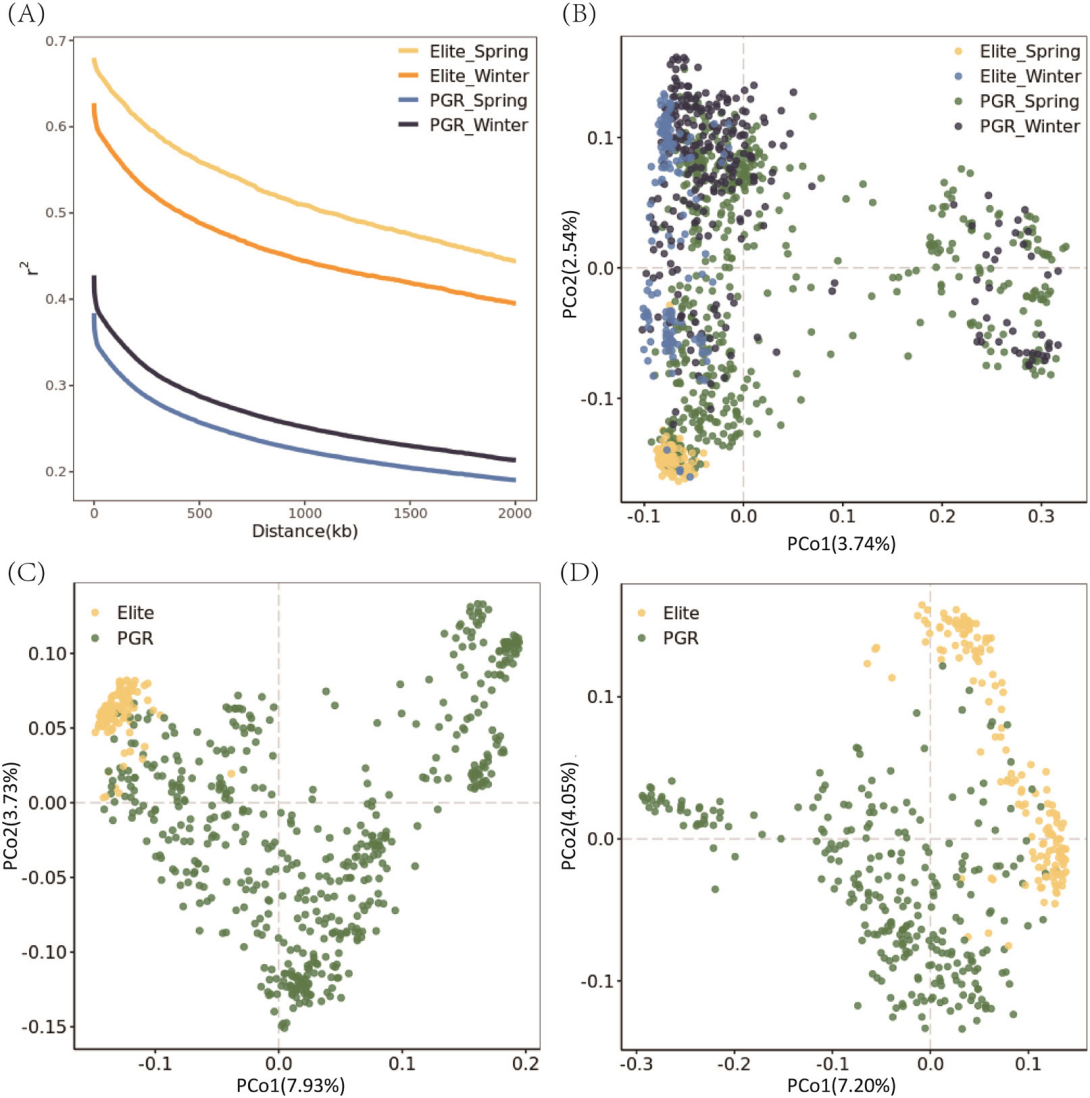
LD decay and genetic diversity analysis for the collection of 1110 barley genotypes. (A) LD (*r*^2^) decay plot as a function of physical distance (kb) of the four subgroups. The principal coordinate analysis (PCoA) is portrayed in a bi-plot of the first two principal coordinates based on pairwise Rogers’ distance matrix for the whole collection (B), the spring population (C), and the winter population (D). PGR, plant genetic resource.

The entire barley collection analyzed in this study comprised 1110 lines that were genotyped using short-read whole-genome skim-sequencing. Read mapping and variant calling against the MorexV3 reference provided a total of 149 380 812 SNPs. Subsequent SNP filtering, conducted on a subgroup basis, resulted in a total of 57 559 591 unique SNP markers meeting criteria of a maximum 10% missing value rate and a minimum 5% minor allele frequency. The markers that passed filtration were then used to calculate the genome-wide mean LD for each corresponding subgroup, separately. At a distance of 2 Mb, LD decayed 1.2- and 1.1-fold faster in the PGR pool than in the elites for spring and winter types, respectively (Fig. 2A). The slower LD decay in the elites may be due to genetic bottlenecks and/or high selection pressures that produce specific linkage between alleles that control specific phenotypes. The differences in LD decay, which mirror the genetic structure and an uneven level of relatedness within the associated panel, pose significant challenges leading to false association inflation in GWAS, thus, demonstrating the need to segregate the entire population into four subgroups.

The markers that passed the quality control process were further imputed and pruned, yielding 4 940 383 unique SNPs. Subsequently, the genetic structure was investigated by applying PCoA based on Rogers’ distance matrix on three levels: the entire collection, only spring barley, and only winter barley. A joint PCoA for the whole set (Fig. 2B) revealed that both PGR_Spring and PGR_Winter subsets covered the same diversity space. In the spring barley population, the elite lines and PGR accessions were clearly separated by the first two PCos, explaining together 11.66% of the total genetic variation (Fig. 2C). Likewise, elites and PGRs showed clear patterns in the winter population with few instances of admixture (Fig. 2D). Within the elite population, which mostly comprises European varieties, distinct grouping among the two growth habit clusters highlights the significant influence of geographical origin on the genetic structure of elite barley varieties (Fig. 2B).

### Traits phenotyped across multiple environments showed substantial heritability

**Fig. 3.**
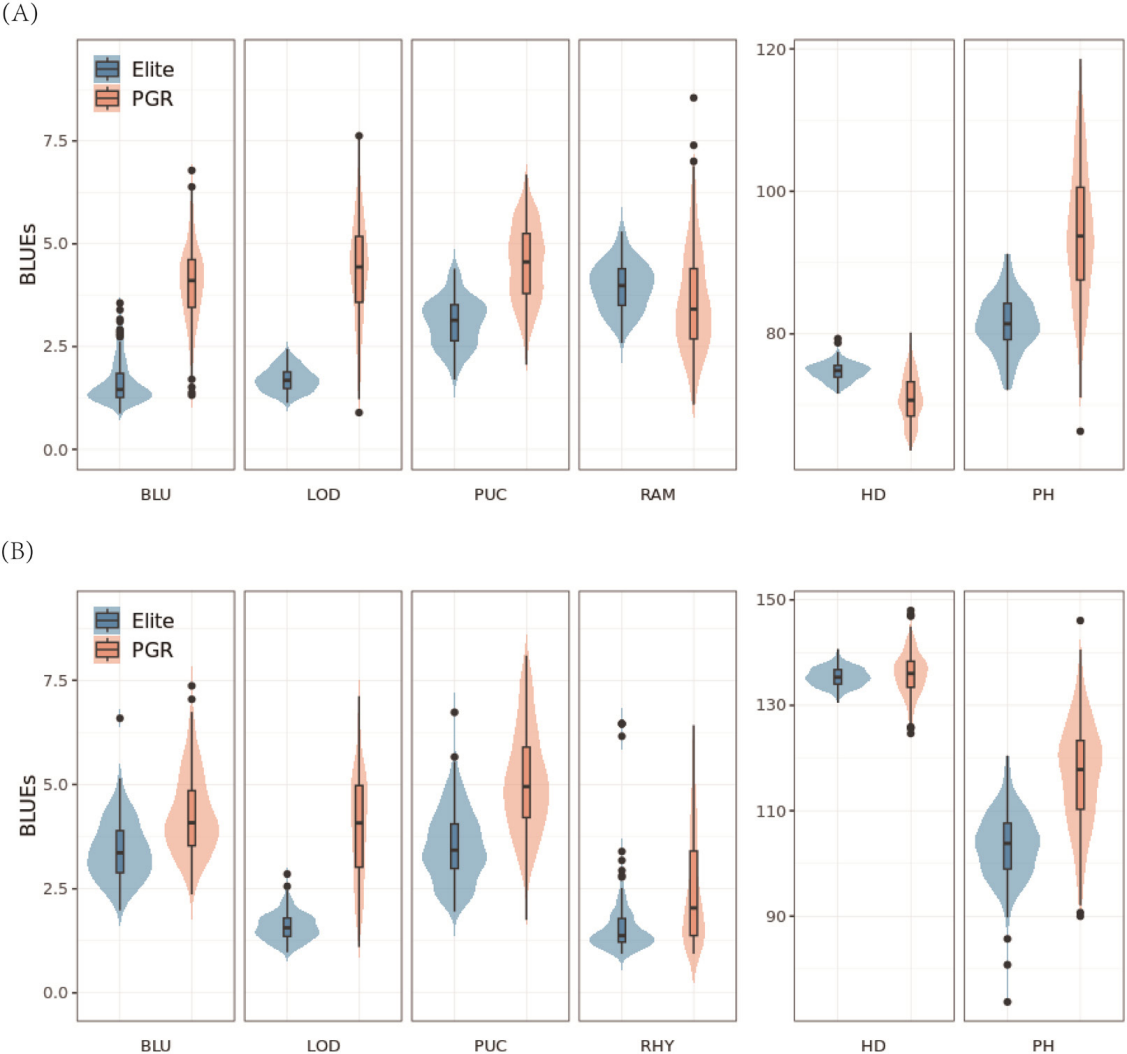
The distribution of best linear unbiased estimates (BLUEs) of the elites and plant genetic resources in spring type (A) and winter type (B). PGR, plant genetic resources; BLU, *Blumeria graminis hordei*; PUC, *Puccinia hordei*; RHY, *Rhynchosporium commune*; RAM, *Ramularia collo-cygni*; HD, heading date; PH, plant height; LOD, lodging.

LMMS combined with rigorous quality assessment were implemented for the phenotypic data recorded for four diseases (PUC, BLU, RAM, and RHY) and three agronomic traits (PH, HD, and LOD). The 1110 genotypes were phenotyped across eight locations over three consecutive years (2020, 2021, and 2022), resulting in a range of 3–22 environments from different year×location combinations. Overall, a wide range of phenotypic variability among the elite and PGR groups for both spring and winter collections was observed, and the adjusted mean values across environments appeared to be normally distributed. The PGRs were more susceptible than the elite materials in spring and winter populations according to the mean severity scores for diseases (Fig. 3; Table 1). Furthermore, contrasting the disease scores of BLU and PUC between elites and PGRs, they appear significantly more pronounced within the spring panel (*P*-value <0.001) compared with the winter panel. For instance, BLU showed reduced disease severity by almost two scoring points in spring as compared with the winter panel. This discrepancy could be attributed to the broader genetic diversity (Fig. 2B) and geographical distribution (Fig. 1) observed in the spring compared with the winter collection.

**Table 1.** ANOVA for the studied traits in spring and winter populations

Growth habit	Trait	σ^2^_*g*_	σ^2^_*g*×*E*_	σ^2^_*e*_	NE	*h* ^2^	CV (%)
Spring	BLU	1.49***	0.98***	2.48***	17	0.91	31.01
	PUC	0.96***	0.84***	3.53*	17	0.88	26.09
	RHY	0.06^NS^	3.40^NS^	0.31***	3	0.05	0.13
	RAM	0.59***	2.25***	2.57***	6	0.54	21.17
	HD	11.53***	8.15***	71.17***	22	0.96	1.36
	PH	90.67***	63.82***	109.74^NS^	13	0.93	5.43
	LOD	2.08***	2.86***	2.70***	17	0.91	19.60
Winter	BLU	0.68***	1.87***	2.93*	11	0.77	17.95
	PUC	1.64***	1.51***	1.52*	12	0.92	12.53
	RHY	1.31***	1.52^NS^	0.66***	10	0.89	15.43
	RAM	2.15E-06***	4.52E-07***	5.56*	5	2.00E-06	54.43
	HD	11.14***	2.73^NS^	36.53***	14	0.95	1.59
	PH	113.50^NS^	7.61E-06^NS^	179.75***	12	0.95	7.78
	LOD	2.17***	3.19*	0.96***	12	0.89	1.68

σ^2^_*g*_, genotypic variance; σ^2^_*g*×*E*_, variance due to genotype by environment interaction; σ^2^_*e*_, variance due to environment; NE, number of environments; *h*^2^, broad-sense heritability; CV, coefficient of variation; BLU, *Blumeria graminis hordei*; PUC, *Puccinia hordei*; RHY, *Rhynchosporium commune*; RAM, *Ramularia collo-cygni*; HD, heading date; PH, plant height; LOD, lodging. *P=0.05 and ***P=0.001; ^NS^, not significant.

Heritability estimates ranged from low (RHY, *h*^2^_spring_=0.05; RAM, *h*^2^_winter_=2.00E-06), to moderate (RAM, *h*^2^_spring_=0.54), to high (from 0.77 to 0.96; Table 1). In order to provide a solid basis for the subsequent association analysis, we omitted the traits exhibiting low heritability (RHY, *h*^2^_spring_=0.05; RAM, *h*^2^_winter_=2.00E-06), since the low heritability is a limiting factor that reduces the power of GWAS to detect associations. The ANOVA showed that the genotypic variance was highly significant (*P*≤0.0001) for all disease traits except for RHY of the spring population, and plant height of the winter population (Table 1). The coefficients of variation (CVs) ranged from 1.36% to 31.01%. In the spring population, environments accounted for the highest proportion of the total variation while genotypes and the genotype×environment interaction were comparatively less than the overall variation for the highly heritable disease traits (BLU, PUC, and RAM), whereas for the winter population, variance components had almost comparable magnitude with few exceptions. The substantial phenotypic variance, in combination with the medium and high heritability, suggests that variation in the disease traits is governed by numerous genetic loci, rendering it suitable for GWAS analysis.

**Fig. 4.**
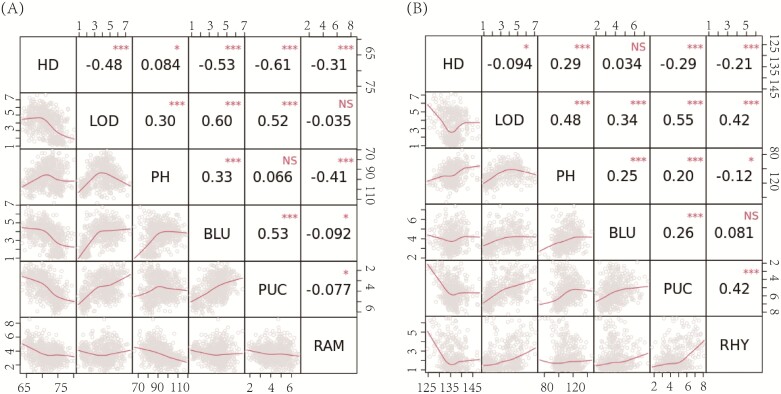
Pearson correlation coefficients (*r*) among traits. (A) Pearson correlation coefficients in the spring barley (A) and winter barley (B) collection. The values above the diagonal represent Pearson’s *r* between traits, and the plots below the diagonal are the scatter plots for the respective traits. BLU, *Blumeria graminis hordei*; PUC, *Puccinia hordei*; RHY, *Rhynchosporium commune*; RAM, *Ramularia collo-cygni*; HD, heading date; PH, plant height; LOD, lodging; NS. not significant; *P<0.05; ***P<0.001.

Pearson’s correlation coefficient was computed to assess the relationship among the studied traits within the spring and winter populations, separately. PUC and BLU were positively correlated (*r*=0.53, *P*<0.001; Fig. 4A) in the spring population. Nevertheless, RAM exhibited a modest negative correlation with both BLU and PUC, implying the independent evolution of these traits and subsequent alterations to their virulence patterns that do not align strongly. For the winter population (Fig. 4B), PUC is positively associated with RHY (*r*=0.42, *P*<0.001) and far less correlated with BLU (0.26, *P*<0.001). This correlation pattern suggests the contribution of common genetic factors influencing the resistance level of the diseases in question.

Disease resistance traits were also found to be correlated with some agronomical traits, indicating an association between plant phenology and disease progression. The correlation between diseases and agronomical traits was more pronounced for most cases in the spring compared with the winter population. In the spring panel, lodging was positively correlated with BLU (*r*=0.60, *P*<0.001) and PUC (*r* =0.52, *P*<0.001), and plant height was positively correlated with BLU (*r*=0.33, *P*<0.001), but negatively correlated with RAM (*r*= –0.41, *P*<0.001). Heading date was negatively correlated with all BLU, PUC, and RAM, with values of –0.53, –0.61, and –0.31, respectively. In the winter population, the most pronounced correlation was observed between LOD and PUC (*r*=0.55, *P*<0.001), while heading date exhibited either negative or non-significant correlations with the disease traits.

### Genome-wide association analysis revealed QTLs associated with disease resistance traits in barley

The genotypic matrix for the entire collection was divided into four subgroups based on the growth habit and germplasm origin. After quality assessment and the pruning process, the final sets of 710 855 SNPs for Elite_Spring, 945 074 SNPs for Elite_Winter, 2 321 327 SNPs for PGR_Spring, and 1 775 972 SNPs for PGR_Winter, evenly distributed across all the seven chromosomes, were retained for the subsequent analysis (Supplementary Fig. S1). Given the pronounced diversity in population profiles (Fig. 2), genome-wide association analysis was performed separately for the four subgroups using GEMMA and BLINK, mitigating the risk of overcorrection by the kinship matrix. The clustering of genotypes within each subgroup (Supplementary Fig. S2) was controlled by considering the kinship matrix (K) and population structure (Q) as covariance. The observed and expected *P*-values for the vast majority of markers matched, with a clear deviation of the observed values from the expected to the right end of the quantile–quantile plot, indicating that the Q+K model provided control for false-positive associations (Supplementary Figs S3–S9).

A total of 932 marker–trait associations (MTAs) were detected for the four disease resistance traits, showing an uneven distribution across all seven chromosomes (Supplementary Figs S3–S9); the individual significant SNPs explained 0.01–47.81% of the phenotypic variation (Supplementary Table S2; Supplementary Fig. S10). These MTAs were subsequently clustered into 49 individual QTLs by assigning them to the previously calculated haplotype blocks in the whole genome defined based on LD. GWAS results for the three agronomic traits were omitted here as they fall outside the scope of this study; however, we will still explore the intriguing possibility of overlapping MTAs, particularly in relation to heading date. The effect of each marker varied from –3.56 to 2.01. It is noteworthy that BLINK utilizes a set of pseudo-quantitative trait nucleotides as co-variables, effectively ensuring that the detected MTAs sit in different LD blocks. This setting leads to the identification of fewer and more spread out MTAs, distinguishing it from traditional LMMs (e.g. GEMMA; Liu *et al.*, 2023, Preprint).

Pearson’s correlation coefficients and a path analysis revealed a positive association between BLU and PUC within the spring barley population (Fig. 4A; Supplementary Fig. S11) and a positive correlation between RHY and PUC within the winter barley population (Fig. 4B; Supplementary Fig. S12). This relationship is most likely to arise from the co-localization between QTLs and genes governing resistance mechanisms against two different diseases. For instance, the QTL *qPUC2.2*, detected in the PGR_Spring panel sand significantly associated with PUC, is co-located with *mlo3* (*mildew resistance locus o 3*), known for its ability to mitigate the infection caused by *Blumeria graminis hordei*, thereby conferring durable resistance. Additionally, the QTLs *qPUC5.1* and *qPUC5.2*, which exhibited significant associations with PUC in both Elite_Spring and PGR_Spring panels, reside within the genomic region of two potential powdery mildew resistance genes: an *mlo-like* gene and an *EDR2-like* (enhanced disease resistance 2-like) gene. Furthermore, *qBLU1.1*, associated with BLU resistance in the Elite_Spring group, aligns with a high-confidence gene annotation encoding rust resistance protein (Rp1). Moreover, the two QTLs *qBLU4.4* in Elite_Spring and *qPUC4.1* in PGR_Spring were significantly associated with BLU and PUC, respectively. Notably, the genomic region of these two QTLs encompass two high-confidence genes: *mlo-h1* (*mlo-homolog1*) and *mlo* gene. The five QTLs, *qRHY1.1*, *qRHY5.4*, *qRHY5.5*, *qRHY5.6*, and *qRHY5.7*, associated with RHY in the Elite_Winter group were identified in the overlap region of previously reported QTLs associated with PUC (Case *et al.*, 2018; Gyawali *et al.*, 2021). These overlapped genes or regions might be a promising target for further functional validation strategies to improve the cross-resistance of barley.

### The accumulative effect of novel and rare resistance alleles enhanced the resistance level in plant genetic resources and elite materials

**Fig. 5.**
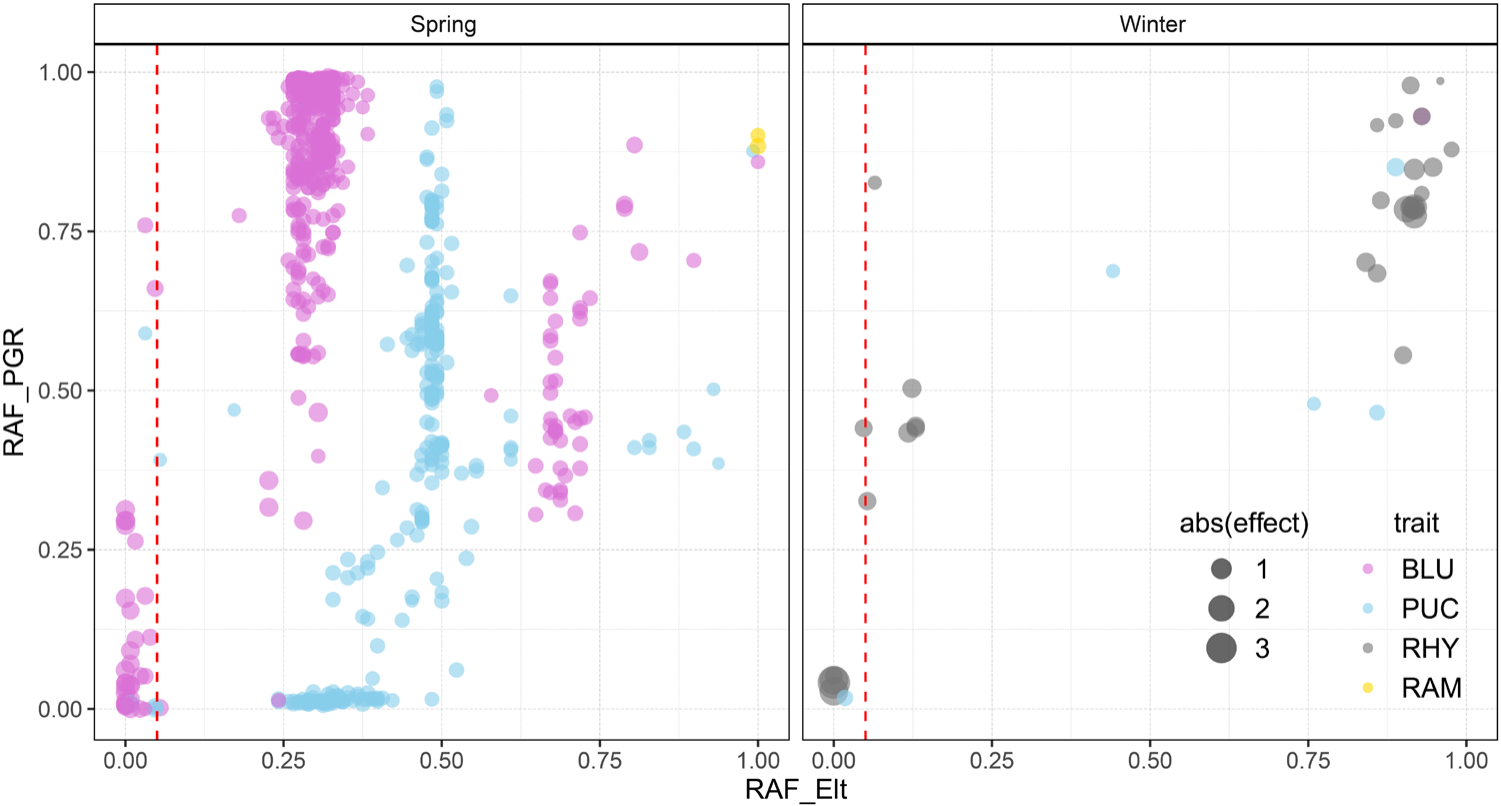
Resistance allele frequency (RAF) of all the significant markers. RAF in elite (*x*-axis) and plant genetic resources (*y*-axis). The plot on the left side shows all the resistance alleles detected in the spring type subgroups; and the plot on the right side shows all the resistance alleles detected in the winter type subgroups. The vertical red dashed line corresponds to the threshold of the RRAs (RAF_Elite_=0.05). PGR, plant genetic resources; BLU, *Blumeria graminis hordei*; PUC, *Puccinia hordei*; RHY, *Rhynchosporium commune*; RAM, *Ramularia collo-cygni*.

Resistance allele mining is a promising approach to take advantage of the PGR gene pools, offering a potential application to enrich the resistant gene portfolio of the elite pool and improve the important agronomic traits of cultivars and/or modern varieties. In this study, we identified the resistance allele of each significant marker. A resistance allele that is absent or rare in elite lines but present in plant genetic resources may hold untapped potential for barley disease resistance improvement. Therefore, we calculated and filtered the frequency for all the resistance alleles within each growth habit, to identify novel resistance alleles (NRAs) which are absent in elite lines [resistant allele frequency (RAF_Elite_)=0], and rare resistance alleles (RRAs) (0<RAF_Elite_<0.05) (Fig. 5).

**Fig. 6.**
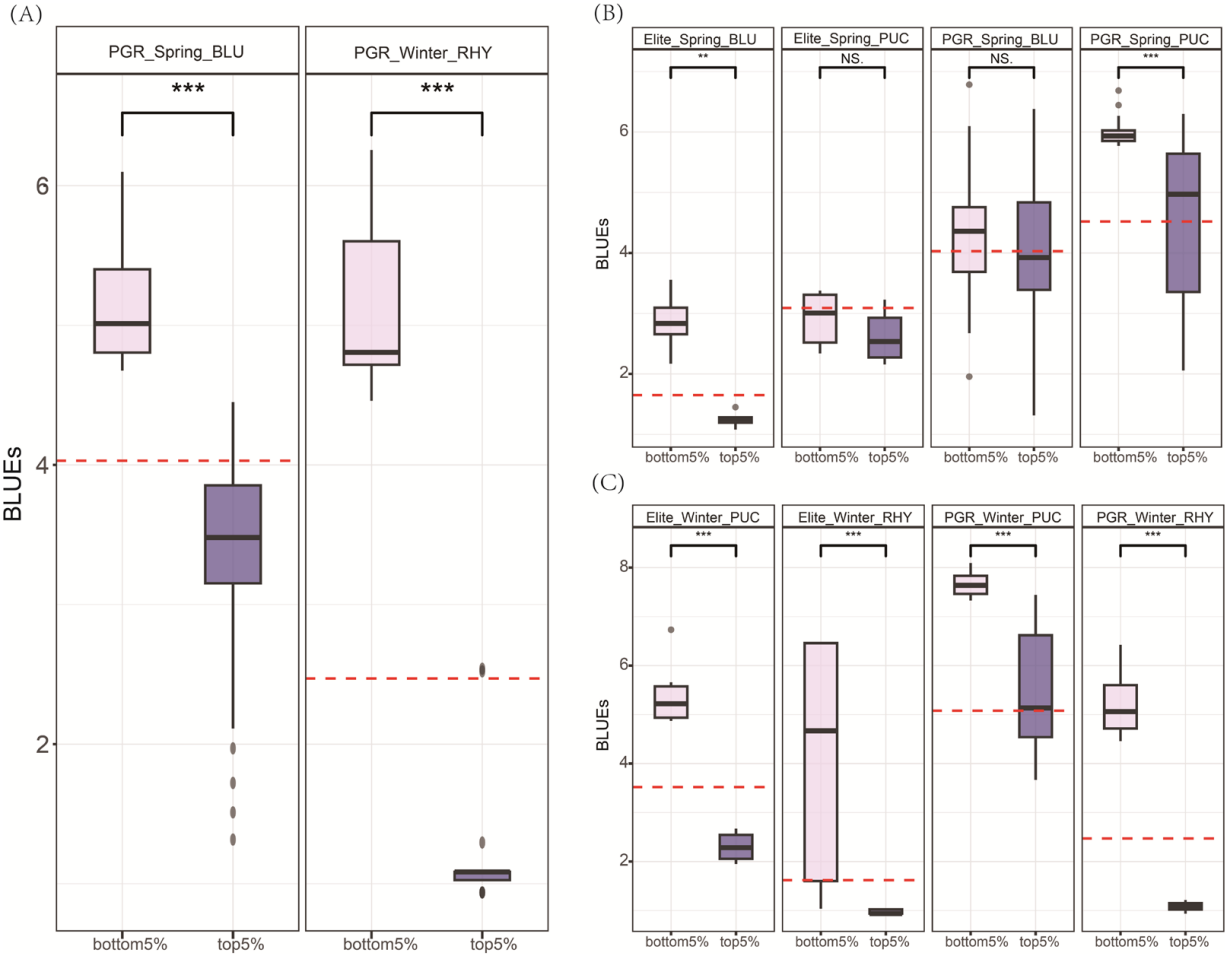
Phenotypic distribution of genotypes containing novel resistance alleles (NRAs) and rare resistance alleles (RRAs). (A) Phenotypic distribution of plant genetic resources that harbored more NRAs (top5%) and fewer NRAs (bottom5%). Phenotypic distribution of genotypes with more RRAs (top5%) and fewer RRAs (bottom5%) in the spring subgroup (B) and the winter subgroup (C). Horizontal lines in the boxplots represent the lower quartile, median, and upper quartile, respectively. The horizontal red dashed line corresponds to the mean value of the trait. PGR, plant genetic resources; BLU, *Blumeria graminis hordei*; PUC, *Puccinia hordei*; RHY, *Rhynchosporium commune*. NS, not significant; **P<0.01; ***P<0.001.

This resulted in 15 NRAs associated with BLU, 18 RRAs associated with BLU, and 4 RRAs associated with PUC (Supplementary Tables S4, S5) within the spring pool. For the winter pool, four NRAs were associated with RHY, one with PUC, and one with RHY (Supplementary Tables S4, S5). Combining alleles, also known as pyramiding or stacking, is pivotal for developing new varieties with enhanced and durable disease resistance. To investigate the effect of pyramiding RAs, we assessed the accumulated contribution of NRAs and RRAs for each genotype on a subgroup basis. We sorted them by count, and subsequently analyzed the phenotypic difference between the 5% of genotypes that harbored the fewest resistance alleles (bottom5%) and the 5% of genotypes with the highest resistance alleles (top5%) using a *t*-test. As shown in Fig. 6A, PGRs carrying higher NRAs exhibited significantly greater resistance to spring BLU and winter RHY compared with PGRs with fewer NRAs. Likewise, for the RRAs within the spring barley (Fig. 6B), the genotypes harboring a greater number of resistance alleles demonstrated significantly higher resistance to BLU and PUC compared with genotypes with fewer resistance alleles in the elite and PGR groups, respectively. This trend held true for the winter population for PUC and RHY in both elites and PGR groups (Fig. 6C). The results suggested that there is a huge potential of accumulating both NRAs and RRAs in future disease resistance breeding programs. For instance, two German spring type PGRs, HOR-22190 and HOR-18711, and one French winter type PGR, HOR-10199, possess the highest counts for the total number of NRAs and RRAs and also exhibited exceptional resistance to powdery mildew and leaf rust (Supplementary Fig. S11). The three potential donors provide a robust groundwork offering the potential for enhanced resilience in future varieties.

## Discussion

### Plant genetic resources are employed to introduce additional diversity into modern barley

Selecting donors that harbor favorable genes and alleles absent in existing varieties is crucial to complement elite recipients (Sanchez *et al.*, 2023). While many programs introgressed valuable qualitative and quantitative alleles from PGRs into elite germplasm (Tarter *et al.*, 2003; Fischer *et al.*, 2010; Schulthess *et al.*, 2022), linkage drag may discourage breeders from using PGRs in their breeding programs, necessitating pre-breeding efforts to retain desirable variability within barley genomes and reduce undesirable genetic load. Previous studies indicated that a substantial 60% of alleles identified in wild barley are absent in cultivated varieties (Ellis *et al.*, 2000). This underscores the significant reservoir of genetic diversity inherent in wild barley, presenting valuable opportunities for diverse breeding initiatives. Therefore, mining the untapped resistance genes in PGR material remains indispensable, offering a continuous incorporation of novel genes or loci to bolster disease resistance within the elite pool. This purpose is best served by the core collection selection strategy that maximizes genetic diversity in the core relative to the entire genebank collections. High-throughput genotyping is instrumental in this process, offering efficient, reliable, and cost-effective tools to collect large-scale sequencing data of entire genebank collections. These tools assist in identifying genetic redundancy in core collections, thus demonstrating their capacity to unambiguously discriminate closely related accessions. In our study, we utilized a core collection of 812 PGRs selected out of the 21 405 barley IPK genebank accessions and 298 European elite materials originating from 60 countries across five continents, representing global diversity in the context of the major barley row type and growth habit subpopulations. However, due to the general lack of adaptation of most PGRs to the current agricultural environments and pathogens (McCouch et al., 2020), it was anticipated that the majority would be susceptible to the adapted pathogen populations. Consequently, a limited proportion of resistant PGRs was identified, underscoring the challenge of relying solely on diversity information rather than covering the phenotypic diversity of several traits in the selection of new germplasm for resistance breeding. Therefore, a trait-customized core collection strategy that balances coverage of both the genotype and phenotype diversity space of a target trait is promising to maximize the success of detecting resistance-related alleles (Schulthess *et al.*, 2022).

### Pleiotropic markers/QTLs confer barley resistance to different diseases

The factors that influence the statistical power of GWAS detection are already well known. While population size plays a crucial role (Alqudah *et al.*, 2020), the ability to detect true GWAS signals can also be improved by a reduction in the correlation between genetic and phenotypic similarity (Myles *et al.*, 2009), coupled with an increase in the frequencies of rare alleles at functional loci (Soto-Cerda and Cloutier, 2012). In this respect, our approach of partitioning the total collection into four distinct subgroups based on the growth habit and germplasm origin decreased, in most cases, the correlation between genetic (Rogers’ distance) and phenotypic distances (Euclidean distance), compared with the larger population based solely on growth habit (Supplementary Table S6). Therefore, we observed an increment in QTL detection power for these four subgroups despite the trade-off with the general power achieved through the initial full-sized population.

In the current study, we detected 932 SNP markers, clustered into 49 QTLs associated with four disease resistance traits, and 474 SNP markers clustered into 23 QTLs associated with three agronomic traits (Supplementary Table S3). Among the 49 disease resistance QTLs, six (PUC resistance: *qPUC1.1*, *qPUC5.1*, and *qPUC5.2*; RHY resistance: *qRHY3.1*, *qRHY3.2*, and *qRHY3.4*) overlapped with previously reported QTLs (Genger *et al.*, 2003; Sayed *et al.*, 2004; Case *et al.*, 2018; Zhang *et al.*, 2020; Gyawali *et al.*, 2021). Fourteen QTLs were co-located with genes annotated to encode the disease resistance proteins of the nucleotide-binding leucine-rich repeat sequence (NBS-LRR) and Toll-interleukin receptor (TIR)-NBS-LRR family. Additionally, seven QTLs were co-located with genes annotated for disease resistance proteins such as resistance gene analog 2 (RGA2), downy mildew regulator RPP13, and RPM1. LRR proteins are resistance proteins encoded by the NBS-LRR gene in plants which plays a vital role in plant defense against many various pathogens (Jones and Jones, 1997). Notably, an LRR protein was identified to be involved in the barley defense mechanism against spot blotch (*Cochliobolus sativus*; Ameen *et al.*, 2020, Preprint). These findings underscore the effectiveness of GWAS in this study in pinpointing alleles associated with resistance to the four diseases, and provide starting points for further genetic and functional analysis.

Additionally, four QTLs associated with PUC also co-located with *mlo*, *mlo-like*, or *mlo-homolog* genes. This observation aligns with the significant positive association observed in Pearson’s correlation coefficient (*r*=0.53, *P*<0.001) and path analysis (Supplementary Fig. S11) between PUC and BLU in the spring panel which has also been reported previously (Spielmeyer *et al.*, 2005; Mago *et al.*, 2011; Lillemo *et al.*, 2018). While there is no evidence that any of the *mlo* alleles confer resistance to barley leaf rust, it has been demonstrated that the presence of various *mlo* alleles (*mlo-1*, *mlo-3*, and *mlo-5*) increases susceptibility to the rice blast fungus (*Magnaporthe grisea*), whereas the wild-type *Mlo* allele confers resistance (Jarosch *et al.*, 1999).

For those aforementioned pleiotropic markers/QTLs, a further validation process is necessary in different genetic backgrounds before using them for the marker-assisted selection in barley disease resistance development, such as the Targeting Induced Local Lesions IN Genomes (TILLING) strategy (Talamè *et al.*, 2008; Szurman-Zubrzycka *et al.*, 2018; Rosignoli *et al.*, 2022), and the development of kompetitve allele-specific PCR (KASP; Rasheed *et al.*, 2016) assays.

### Co-localization of disease resistance and agronomy trait QTLs

Considering together the GWAS results, it appears that three putative QTLs associated with agronomic traits overlapped with those identified from disease resistance traits. *qHD2.1*, associated with the heading date, shared five and one common significant markers with *qPUC2.1* and *qPUC2.2*, respectively. These six common markers are enriched within ~0.3 Mb on the short arm of chromosome 2. Furthermore, the three aforementioned QTLs were co-mapped with the gene *Photoperiod-H1* (*Ppd-H1*) which promotes flowering under long-day conditions (Turner *et al.*, 2005). Additionally, *qHD5.2*, also found to be associated with the heading date, co-localized with *qPUC5.1*. Those observations suggest a strategic plant response given that delayed heading allows plants to evade disease infection through spatial or temporal adjustments. This dynamic alignment reflects the intricate interplay between phenology shifts and disease resistance, enhancing our understanding of the nuanced strategies employed by plants to thrive in complex environments. The observed negative correlation (*r*= –0.66, *P*<0.001) and the path analysis (Supplementary Fig. S11) between the heading date and PUC within the spring panel further support these results. A similar pleiotropic effect has been reported in oat (Portyanko *et al.*, 2005; Acevedo *et al.*, 2010), suggesting a pivotal role for these regions exerting multifaceted control. Subsequent studies are needed to disentangle the effect of disease traits from the agronomy values and dissect these putative QTLs more comprehensively, given that a QTL for one trait can often be antagonistically coupled with other breeding targets.

Nevertheless, none of the QTLs detected for plant height and lodging overlapped with disease-resistant QTLs. The closest signal was detected for heading date on chromosome 1 (*qHD1.2*), ~3 Mb downstream of *qBLU1.1*. These two QTLs reside at a relatively large distance from each other, greater than the LD decay threshold we employed; hence, they are considered different loci. The absence of overlapping QTLs among these traits suggests that distinct genetic mechanisms may govern disease susceptibility and the two agronomic traits in the tested material.

### Novel and rare resistance allele accumulation empowered the selection of promising plant genetic resources for future barley disease resistance improvement

Our study showed that the phenotypic variability of the four assessed disease traits was governed by many minor effect QTLs (Supplementary Fig. S13). Thus, a strategy of combining those minor effect resistance alleles could provide durable resistance (Parlevliet and Zadoks, 1977). Resistance alleles inherited from the donors and subsequently fixed in modern varieties formed the basis of barley disease resistance, harnessing favorable allele effects for enhanced genetic gain. Resistance alleles, either absent or present at low frequencies in elite groups, are still underutilized in modern breeding programs and should be recognized as essential elements for genetic enhancement of disease resistance. In this study, we identified 15 NRAs and 18 RRAs in the spring group, as well as four NRAs and two RRAs in the winter group. Also there was a significant trend where disease scores consistently decreased with the accumulation of both NRAs and RRAs.

Combining several resistance-conferring alleles in a variety, known as gene pyramiding or stacking, should strengthen the defensive barrier for one or more pathogens, thereby increasing the challenge for them to overcome (Dong and Ronald, 2019). The insights gleaned from the aforementioned findings will serve as a foundation to identify superior performing PGRs that harbored the greatest number of novel and rare resistance alleles. Given that phenotypic selection alone can be misleading due to human errors (Svensson, 2023), selection based on the combination of phenotypic performance with the total number of target alleles will lead to target crosses with desired traits, ultimately contributing to the success of the breeding programs (Peleman and Van der Voort, 2003). This strategy has been widely used in the selection of resistant donors in barley (Hautsalo *et al.*, 2021; Esmail *et al.*, 2023), wheat (Luo *et al.*, 2021), and cotton (Mei *et al.*, 2013; Dai *et al.*, 2019). Our study shed light on the potential benefits of accumulating together NRAs and RRAs in a few accessions, showcasing their ability to confer higher resistance to diseases. For instance, two German spring type PGRs, HOR-22190 and HOR-18711, and one French winter type PGR, HOR-10199, stood out by possessing the highest counts for the total number of NRAs and RRAs and exhibited exceptional resistance to two diseases (Supplementary Fig. S11). These potential donors provide a robust grounding for targeted pre-breeding goals and offer the potential for enhanced resilience in future varieties. To substantiate the effectiveness of novel and rare disease resistance alleles in variety development, doubled haploid populations have been constructed and will undergo rigorous evaluation in field conditions, confirming their potential for broad-spectrum resistance capabilities and achieving durable resistance.

### The follow-up strategy for barley resistance breeding: towards an integrated approach

Divergent approaches in introgression strategies often revolve around the distinctive contributions of genes or loci to phenotypic variation, so-called major or minor effect resistance (R) genes or alleles. Major effect R genes derived from PGRs emerge as a robust strategy to make crops more resistant (Hospital and Charcosset, 1997; Servin *et al.*, 2004; Dormatey *et al.*, 2020; Abdelraheem *et al.*, 2024; Attamah *et al.*, 2024). However, the introgression of single major R genes into new varieties carries the risk of being overcome by rapidly evolving pathogens. Therefore, it would be ideal, but challenging, to stack multiple major genes and, hence, minimize the likelihood of resistance breakdown. In contrast, while major genes offer immediate and dramatic phenotypic changes, the cumulative enhancement of resistance traits through minor allele introgression often exerts less selection pressure on pathogen populations, making them less likely to induce pathogen adaptation, fostering resilient and enduring resistance in new varieties. However, the polygenic nature of minor alleles requires complicated breeding strategies to strike a balance between sustained resistance and the challenges of managing multiple small effect alleles. Therefore, embracing a comprehensive approach that combines major and minor genes that have minimal adverse epistasis effects enables breeders to respond to evolution of the pathogen. This advocated approach requires integrating QTLome-based methods (such as marker-assisted selection, cloning, editing, and genetic engineering) with genomic selection-based approaches. By doing so, breeders can effectively utilize the gene-for-gene concept, which leads to specific interactions between a plant’s resistance gene and a pathogen’s avirulence gene. Major QTLs can significantly influence the expression of resistance traits, acting similarly to major R genes but often with a broader impact on the plant’s overall genetic makeup. The concept of the QTLome, which encompasses the totality of QTLs associated with the targeted disease resistance in a particular species, provides a comprehensive framework for utilizing these genetic resources (Salvi and Tuberosa, 2015). By integrating major QTLs into breeding programs alongside major and minor R genes, breeders can develop more robust and adaptable disease resistance strategies, enhancing both vertical and horizontal resistance in new crop varieties.

On the other hand, to address the potential challenges of a significant performance gap between the PGR donors and elites, it is important to account for the complementarity between the donors and the elite recipient before the crossing process. Allier *et al.* (2020) proposed a Usefulness Criterion Parental Contribution (UCPC) approach to predict the interest of crosses between donors and the elite recipients, and it is necessary to consider a buffer population before the integration process, also referred to as a bridging population (Allier *et al.*, 2020). Subsequently, the best progeny identified through the bridging process are introduced into the elite breeding population using several breeding methods, including recurrent selection and genomic selection. These proven approaches are reliable for accumulating alleles with relatively large and small effects, making them effective for achieving breeding objectives (Nelson *et al.*, 2018).

While new technologies hold promise, a pragmatic balance must be struck between field management and the basics of plant breeding. The diversity of pathogens and their ongoing evolution makes resistance breeding nontrivial. Despite efforts, agrochemicals often lose effectiveness due to pathogen resistance, leading to increased crop susceptibility and reduced agricultural productivity. Therefore, an eco-evolutionary alternative is necessary by utilizing intraspecific diversity employing cultivar mixtures or multi-lines with different levels of resistance, arrayed with random patterns (Mundt, 2002). This strategy not only reduces plant disease epidemics in the short term but also hinders pathogen evolution in the long run. It may thus minimize the overall impact of disease outbreaks and forestall the appearance of fungicide insensitivity. All this will contribute to improving the sustainability of agriculture worldwide (Papaïx *et al.*, 2011).

## Supplementary data

The following supplementary data are available at *JXB* online.

Table S1. List of 1110 genotypes used in this study.

Table S2. Summary of all the QTLs of four disease resistance traits.

Table S3. Summary of all the QTLs of three agronomy traits.

Table S4. Summary of all the novel resistance alleles.

Table S5. Summary of all the rare resistance alleles.

Table S6. Mantel test for comparison of distance matrices.

Fig. S1. The marker distribution and density.

Fig. S2. Heatmap of pairwise kinship matrix.

Figs S3–S9. Quantile–quantile plot (left side) and Manhattan plots (right side) of the genome association analysis results.

Fig. S10. Histogram of the phenotypic variation explained by QTLs associated with four diseases traits.

Fig. S11. Path analysis between heading date and other disease resistance traits in spring barley.

Fig. S12. Path analysis between heading date and other disease resistance traits in winter barley.

Fig. S13. Phenotype distribution of disease score with the accumulation of the total number of rare resistance alleles (RRAs) and novel resistance alleles (NRAs) of plant genetic resources in spring and winter types.

erae283_suppl_Supplementary_Tables_S1-S6

erae283_suppl_Supplementary_Figures_S1-S13

## Data Availability

All the sequence data collected in this study have been deposited at the European Nucleotide Archive (ENA, https://www.ebi.ac.uk/ena/) under BioProjects PRJEB53924 (Illumina resequencing data). Accession codes and best linear unbiased estimates (BLUEs) for individual genotypes are listed in Supplementary Table S1.

## References

[CIT0001] Ababa G , Kesho A, Tadesse Y, Amare D. 2023. Reviews of taxonomy, epidemiology, and management practices of the barley scald (*Rhynchosporium graminicola*) disease. Heliyon 9, e14315. 36938428 10.1016/j.heliyon.2023.e14315 PMC10018571

[CIT0002] Abdelraheem A , Zhu Y, Zeng L, Stetina S, Feng C, Wheeler T, Zhang J. 2024. Identification of new genetic sources of resistance to bacterial blight race 18 in diploid Asiatic cotton and resistance transfer to tetraploid Upland cotton (*Gossypium hirsutum*). Euphytica 220, 85.

[CIT0003] Acevedo M , Jackson EW, Chong J, Rines HW, Harrison S, Bonman JM. 2010. Identification and validation of quantitative trait loci for partial resistance to crown rust in oat. Phytopathology 100, 511–521. 20373973 10.1094/PHYTO-100-5-0511

[CIT0004] Allier A , Teyssèdre S, Lehermeier C, Moreau L, Charcosset A. 2020. Optimized breeding strategies to harness genetic resources with different performance levels. BMC Genomics 21, 349. 32393177 10.1186/s12864-020-6756-0 PMC7216646

[CIT0005] Alqudah AM , Sallam A, Stephen Baenziger P, Börner A. 2020. GWAS: fast-forwarding gene identification and characterization in temperate cereals: lessons from barley—a review. Journal of Advanced Research 22, 119–135. 31956447 10.1016/j.jare.2019.10.013 PMC6961222

[CIT0006] Ameen G , Solanki S, Drader T, Sager-Bittara L, Steffenson B, Kleinhofs A, Vogiatzis C, Brueggeman RS. 2020. *rcs5*-mediated spot blotch resistance in barley is conferred by wall-associated kinases that resist pathogen manipulation. bioRxiv. doi: 10.1101/2020.04.13.040238. [Preprint].

[CIT0007] Anscombe FJ , Tukey JW. 1963. The examination and analysis of residuals. Technometrics 5, 141–160.

[CIT0008] Attamah P , Kusi F, Kena AW, Awuku FJ, Lamini S, Mensah G, Zackaria M, Owusu EY, Akromah R. 2024. Pyramiding aphid resistance genes into the elite cowpea variety, Zaayura, using marker-assisted backcrossing. Heliyon 10, e31976. 38868054 10.1016/j.heliyon.2024.e31976 PMC11167344

[CIT0009] Browning BL , Zhou Y, Browning SR. 2018. A one-penny imputed genome from next-generation reference panels. American Journal of Human Genetics 103, 338–348. 30100085 10.1016/j.ajhg.2018.07.015 PMC6128308

[CIT0010] Bundessortenamt. 2000. Richtlinien für die Durchführung von landwirtschaftlichen Wertprüfungen und Sortenversuchen. https://www.bundessortenamt.de/bsa/sorten/sortenzulassung/richtlinien-fuer-die-durchfuehrung-von-landwirtschaftlichen-wertpruefungen-und-sortenversuchen.

[CIT0011] Butler DG , Cullis BR, Gilmour AR, Gogel BJ, Thompson R. 2023. ASReml estimates variance components under a general linear mixed model. https://asreml.kb.vsni.co.uk/wp-content/uploads/sites/3/2018/07/ASReml-Package.pdf

[CIT0012] Camenzind M , Koller T, Armbruster C, Jung E, Brunner S, Herren G, Keller B. 2024. Breeding for durable resistance against biotrophic fungal pathogens using transgenes from wheat. Molecular Breeding 44, 8. 38263979 10.1007/s11032-024-01451-2 PMC10803697

[CIT0013] Case AJ , Bhavani S, Macharia G, Steffenson BJ. 2018. Genome-wide association study of stem rust resistance in a world collection of cultivated barley. Theoretical and Applied Genetics 131, 107–126. 29177535 10.1007/s00122-017-2989-y

[CIT0014] Chełkowski J , Tyrka M, Sobkiewicz A. 2003. Resistance genes in barley (*Hordeum vulgare* L.) and their identification with molecular markers. Journal of Applied Genetics 44, 291–309. 12923305

[CIT0015] Dai P , Miao Y, He S, et al. 2019. Identifying favorable alleles for improving key agronomic traits in upland cotton. BMC Plant Biology 19, 138. 30975072 10.1186/s12870-019-1725-y PMC6458685

[CIT0016] Daly MJ , Rioux JD, Schaffner SF, Hudson TJ, Lander ES. 2001. High-resolution haplotype structure in the human genome. Nature Genetics 29, 229–232. 11586305 10.1038/ng1001-229

[CIT0017] Dong OX , Ronald PC. 2019. Genetic engineering for disease resistance in plants: recent progress and future perspectives. Plant Physiology 180, 26–38. 30867331 10.1104/pp.18.01224 PMC6501101

[CIT0018] Dormatey R , Sun C, Ali K, Coulter JA, Bi Z, Bai J. 2020. Gene pyramiding for sustainable crop improvement against biotic and abiotic stresses. Agronomy 10, 1255.

[CIT0019] Dracatos PM , Lu J, Sánchez‐Martín J, Wulff BBH. 2023. Resistance that stacks up: engineering rust and mildew disease control in the cereal crops wheat and barley. Plant Biotechnology Journal 21, 1938–1951. 37494504 10.1111/pbi.14106 PMC10502761

[CIT0020] Ellis RP , Forster BP, Robinson D, Handley LL, Gordon DC, Russell JR, Powell W. 2000. Wild barley: a source of genes for crop improvement in the 21st century? Journal of Experimental Botany 51, 9–17. 10938791

[CIT0021] Epskamp S. 2015. semPlot: unified visualizations of structural equation models. Structural Equation Modeling 22, 474–483.

[CIT0022] Esmail SM , Jarquín D, Börner A, Sallam A. 2023. Genome-wide association mapping highlights candidate genes and immune genotypes for net blotch and powdery mildew resistance in barley. Computational and Structural Biotechnology Journal 21, 4923–4932. 37867969 10.1016/j.csbj.2023.10.014 PMC10585327

[CIT0023] Fischer S , Melchinger AE, Korzun V, Wilde P, Schmiedchen B, Möhring J, Piepho H-P, Dhillon BS, Würschum T, Reif JC. 2010. Molecular marker assisted broadening of the Central European heterotic groups in rye with Eastern European germplasm. Theoretical and Applied Genetics 120, 291–299. 19669632 10.1007/s00122-009-1124-0

[CIT0024] Genger RK , Williams KJ, Raman H, Read BJ, Wallwork H, Burdon JJ, Brown AHD. 2003. Leaf scald resistance genes in *Hordeum vulgare* and *Hordeum vulgare* ssp. *spontaneum*: parallels between cultivated and wild barley. Australian Journal of Agricultural Research 54, 1335–1342.

[CIT0025] Gou M , Balint‐Kurti P, Xu M, Yang Q. 2023. Quantitative disease resistance: multifaceted players in plant defense. Journal of Integrative Plant Biology 65, 594–610. 36448658 10.1111/jipb.13419

[CIT0026] Gyawali S , Mamidi S, Chao S, Bhardwaj SC, Shekhawat PS, Selvakumar R, Gangwar OP, Verma RPS. 2021. Genome-wide association studies revealed novel stripe rust resistance QTL in barley at seedling and adult-plant stages. Euphytica 217, 3.

[CIT0027] Hautsalo J , Novakazi F, Jalli M, et al. 2021. Pyramiding of scald resistance genes in four spring barley MAGIC populations. Theoretical and Applied Genetics 134, 3829–3843. 34350474 10.1007/s00122-021-03930-y PMC8580920

[CIT0028] Havis ND , Brown JKM, Clemente G, et al. 2015. *Ramularia collo-cygni*—an emerging pathogen of barley crops. Phytopathology 105, 895–904. 25626073 10.1094/PHYTO-11-14-0337-FI

[CIT0029] Hill WG , Robertson A. 1968. Linkage disequilibrium in finite populations. Theoretical and Applied Genetics 38, 226–231. 24442307 10.1007/BF01245622

[CIT0030] Holm S. 1979. A simple sequentially rejective multiple test procedure. Scandinavian Journal of Statistics 6, 65–70.

[CIT0031] Horsley RD , Hochhalter M. 2004. BARLEY | agronomy. In: Wrigley C, ed. Encyclopedia of grain science. Amsterdam: Elsevier, 38–46.

[CIT0032] Hospital F , Charcosset A. 1997. Marker-assisted introgression of quantitative trait loci. Genetics 147, 1469–1485. 9383086 10.1093/genetics/147.3.1469 PMC1208267

[CIT0033] Huang M , Liu X, Zhou Y, Summers RM, Zhang Z. 2019. BLINK: a package for the next level of genome-wide association studies with both individuals and markers in the millions. GigaScience 8, giy154. 30535326 10.1093/gigascience/giy154 PMC6365300

[CIT0034] Jarosch B , Kogel KH, Schaffrath U. 1999. The ambivalence of the barley *Mlo* locus: mutations conferring resistance against powdery mildew (*Blumeria graminis* f. sp. *hordei*) enhance susceptibility to the rice blast fungus *Magnaporthe grisea*. Molecular Plant-Microbe Interactions 12, 508–514.

[CIT0035] Jayakodi M , Lu Q, Pidon H, et al. 2024. Adaptive diversification through structural variation in barley. bioRxiv. doi: 10.1101/2024.02.14.580266. [Preprint].

[CIT0036] Jones DA , Jones JDG. 1997. The role of leucine-rich repeat proteins in plant defenses. Advances in Botanical Research 24, 89–167.

[CIT0037] Khoury C , Laliberté B, Guarino L. 2010. Trends in *ex situ* conservation of plant genetic resources: a review of global crop and regional conservation strategies. Genetic Resources and Crop Evolution 57, 625–639.

[CIT0038] Kusch S , Panstruga R. 2017. *mlo*-based resistance: an apparently universal ‘Weapon’ to defeat powdery mildew disease. Molecular Plant-Microbe Interactions 30, 179–189. 28095124 10.1094/MPMI-12-16-0255-CR

[CIT0039] Langridge P. 2018. Economic and academic importance of barley. In: Stein N, Muehlbauer GJ, eds. The barley genome. Cham: Springer, 1–10.

[CIT0040] Li H. 2011. A statistical framework for SNP calling, mutation discovery, association mapping and population genetical parameter estimation from sequencing data. Bioinformatics 27, 2987–2993. 21903627 10.1093/bioinformatics/btr509 PMC3198575

[CIT0041] Li H. 2018. Minimap2: pairwise alignment for nucleotide sequences. Bioinformatics 34, 3094–3100. 29750242 10.1093/bioinformatics/bty191 PMC6137996

[CIT0042] Lillemo M , Singh RP, Huerta-Espino J, Chen XM, He ZH, Brown JKM. 2018. Leaf rust resistance gene *LR34* is involved in powdery mildew resistance of CIMMYT bread wheat line Saar. In: Buck HT, Nisi JE, Salomón N, eds. Wheat production in stressed environments. Dordrecht: Springer, 97–102.

[CIT0043] Lipka AE , Tian F, Wang Q, Peiffer J, Li M, Bradbury PJ, Gore MA, Buckler ES, Zhang Z. 2012. GAPIT: genome association and prediction integrated tool. Bioinformatics 28, 2397–2399. 22796960 10.1093/bioinformatics/bts444

[CIT0044] Liu F , Zhang J, Zhao Y, Schmidt RH, Mascher M, Reif JC, Jiang Y. 2023. A comprehensive overview and benchmarking analysis of fast algorithms for genome-wide association studies. bioRxiv. doi: 10.1101/2023.12.05.570105. [Preprint].

[CIT0045] Luo J , Li S, Xu J, Yan L, Ma Y, Xia L. 2021. Pyramiding favorable alleles in an elite wheat variety in one generation by CRISPR-Cas9-mediated multiplex gene editing. Molecular Plant 14, 847–850. 33812982 10.1016/j.molp.2021.03.024

[CIT0046] Mago R , Tabe L, McIntosh RA, et al. 2011. A multiple resistance locus on chromosome arm 3BS in wheat confers resistance to stem rust (*Sr2*), leaf rust (*Lr27*) and powdery mildew. Theoretical and Applied Genetics 123, 615–623. 21573954 10.1007/s00122-011-1611-y

[CIT0047] Martin M. 2011. Cutadapt removes adapter sequences from high-throughput sequencing reads. EMBnet Journal 17, 10.

[CIT0048] Mascher M , Wicker T, Jenkins J, et al. 2021. Long-read sequence assembly: a technical evaluation in barley. The Plant Cell 33, 1888–1906. 33710295 10.1093/plcell/koab077 PMC8290290

[CIT0049] McCouch S , Navabi ZK, Abberton M, et al. 2020. Mobilizing crop biodiversity. Molecular Plant 13, 1341–1344. 32835887 10.1016/j.molp.2020.08.011

[CIT0050] Mei H , Zhu X, Zhang T. 2013. Favorable QTL alleles for yield and its components identified by association mapping in Chinese upland cotton cultivars. PLoS One 8, e82193. 24386089 10.1371/journal.pone.0082193 PMC3873261

[CIT0051] Miedaner T , Korzun V. 2012. Marker-assisted selection for disease resistance in wheat and barley breeding. Phytopathology 102, 560–566. 22568813 10.1094/PHYTO-05-11-0157

[CIT0052] Milner SG , Jost M, Taketa S, et al. 2019. Genebank genomics highlights the diversity of a global barley collection. Nature Genetics 51, 319–326. 30420647 10.1038/s41588-018-0266-x

[CIT0053] Mundt CC. 2002. Use of multiline cultivars and cultivar mixtures for disease management. Annual Review of Phytopathology 40, 381–410. 10.1146/annurev.phyto.40.011402.113723 12147765

[CIT0054] Mundt CC. 2014. Durable resistance: a key to sustainable management of pathogens and pests. Infection, Genetics and Evolution 27, 446–455. 10.1016/j.meegid.2014.01.011 PMC4117828 24486735

[CIT0055] Myles S , Peiffer J, Brown PJ, Ersoz ES, Zhang Z, Costich DE, Buckler ES. 2009. Association mapping: critical considerations shift from genotyping to experimental design. The Plant Cell 21, 2194–2202. 19654263 10.1105/tpc.109.068437 PMC2751942

[CIT0056] Nelson R , Wiesner-Hanks T, Wisser R, Balint-Kurti P. 2018. Navigating complexity to breed disease-resistant crops. Nature Reviews. Genetics 19, 21–33. 10.1038/nrg.2017.82 29109524

[CIT0057] Oksanen J , Simpson GL, Blanchet FG, et al. 2022. vegan: community ecology package. https://cran.r-project.org/web/packages/vegan/index.html. Accessed August 2023.

[CIT0058] Oppermann M , Weise S, Dittmann C, Knüpffer H. 2015. GBIS: the information system of the German Genebank. Database 2015, bav021. 25953079 10.1093/database/bav021 PMC4423411

[CIT0059] Papaïx J , Goyeau H, Du Cheyron P, Monod H, Lannou C. 2011. Influence of cultivated landscape composition on variety resistance: an assessment based on wheat leaf rust epidemics. New Phytologist 191, 1095–1107. 21585391 10.1111/j.1469-8137.2011.03764.x

[CIT0060] Paradis E , Schliep K. 2019. ape 5.0: an environment for modern phylogenetics and evolutionary analyses in R. Bioinformatics 35, 526–528. 30016406 10.1093/bioinformatics/bty633

[CIT0061] Park RF , Golegaonkar PG, Derevnina L, Sandhu KS, Karaoglu H, Elmansour HM, Dracatos PM, Singh D. 2015. Leaf rust of cultivated barley: pathology and control. Annual Review of Phytopathology 53, 565–589. 10.1146/annurev-phyto-080614-120324 26047566

[CIT0062] Parlevliet JE , Zadoks JC. 1977. The integrated concept of disease resistance: a new view including horizontal and vertical resistance in plants. Euphytica 26, 5–21.

[CIT0063] Peleman JD , Van der Voort JR. 2003. Breeding by design. Trends in Plant Science 8, 330–334. 12878017 10.1016/S1360-1385(03)00134-1

[CIT0064] Portyanko VA , Chen G, Rines HW, Phillips RL, Leonard KJ, Ochocki GE, Stuthman DD. 2005. Quantitative trait loci for partial resistance to crown rust, *Puccinia coronata*, in cultivated oat, *Avena sativa* L. Theoretical and Applied Genetics 111, 313–324. 15918009 10.1007/s00122-005-2024-6

[CIT0065] Purcell S , Neale B, Todd-Brown K, et al. 2007. PLINK: a tool set for whole-genome association and population-based linkage analyses. American Journal of Human Genetics 81, 559–575. 17701901 10.1086/519795 PMC1950838

[CIT0066] Rasheed A , Wen W, Gao F, et al. 2016. Development and validation of KASP assays for genes underpinning key economic traits in bread wheat. Theoretical and Applied Genetics 129, 1843–1860. 27306516 10.1007/s00122-016-2743-x

[CIT0067] Rosignoli S , Cosenza F, Moscou MJ, Civolani L, Musiani F, Forestan C, Milner SG, Savojardo C, Tuberosa R, Salvi S. 2022. Cloning the barley *nec3* disease lesion mimic mutant using complementation by sequencing. The Plant Genome 15, e20187. 35302294 10.1002/tpg2.20187 PMC12807410

[CIT0068] Rosseel Y. 2012. lavaan: an R package for structural equation modeling. Journal of Statistical Software 48, doi:10.18637/jss.v048.i02

[CIT0069] Salvi S , Tuberosa R. 2015. The crop QTLome comes of age. Current Opinion in Biotechnology 32, 179–185. 25614069 10.1016/j.copbio.2015.01.001

[CIT0070] Sanchez D , Sadoun SB, Mary-Huard T, Allier A, Moreau L, Charcosset A. 2023. Improving the use of plant genetic resources to sustain breeding programs’ efficiency. Proceedings of the National Academy of Sciences, USA 120, e2205780119. 10.1073/pnas.2205780119 PMC10083577 36972431

[CIT0071] Sayed H , Backes G, Kayyal H, Yahyaoui A, Ceccarelli S, Grando S, Jahoor A, Baum M. 2004. New molecular markers linked to qualitative and quantitative powdery mildew and scald resistance genes in barley for dry areas. Euphytica 135, 225–228.

[CIT0072] Schulthess AW , Kale SM, Liu F, et al. 2022. Genomics-informed prebreeding unlocks the diversity in genebanks for wheat improvement. Nature Genetics 54, 1544–1552. 36195758 10.1038/s41588-022-01189-7

[CIT0073] Servin B , Martin OC, Mézard M, Hospital F. 2004. Toward a theory of marker-assisted gene pyramiding. Genetics 168, 513–523. 15454561 10.1534/genetics.103.023358 PMC1448128

[CIT0074] Shim H , Chasman DI, Smith JD, Mora S, Ridker PM, Nickerson DA, Krauss RM, Stephens M. 2015. A multivariate genome-wide association analysis of 10 LDL subfractions, and their response to statin treatment, in 1868 Caucasians. PLoS One 10, e0120758. 25898129 10.1371/journal.pone.0120758 PMC4405269

[CIT0075] Soto-Cerda BJ , Cloutier S. 2012. Association mapping in plant genomes. In: Caliskan M, ed. Genetic diversity in plants. InTech, 29–54.

[CIT0076] Spielmeyer W , McIntosh RA, Kolmer J, Lagudah ES. 2005. Powdery mildew resistance and *Lr34/Yr18* genes for durable resistance to leaf and stripe rust cosegregate at a locus on the short arm of chromosome 7D of wheat. Theoretical and Applied Genetics 111, 731–735. 15965649 10.1007/s00122-005-2058-9

[CIT0077] Svensson EI. 2023. Phenotypic selection in natural populations: what have we learned in 40 years? Evolution 77, 1493–1504. 37105948 10.1093/evolut/qpad077

[CIT0078] Szurman-Zubrzycka ME , Zbieszczyk J, Marzec M, et al. 2018. *Hor*TILLUS—a rich and renewable source of induced mutations for forward/reverse genetics and pre-breeding programs in barley (*Hordeum vulgare* L.). Frontiers in Plant Science 9, 216. 29515615 10.3389/fpls.2018.00216 PMC5826354

[CIT0079] Talamè V , Bovina R, Sanguineti MC, Tuberosa R, Lundqvist U, Salvi S. 2008. TILLMore, a resource for the discovery of chemically induced mutants in barley. Plant Biotechnology Journal 6, 477–485. 18422888 10.1111/j.1467-7652.2008.00341.x

[CIT0080] Tarter JA , Goodman MM, Holland JB. 2003. Testcross performance of semiexotic inbred lines derived from latin american maize accessions. Crop Science 43, 2272–2278.

[CIT0081] Turner A , Beales J, Faure S, Dunford RP, Laurie DA. 2005. The pseudo-response regulator *Ppd-H1* provides adaptation to photoperiod in barley. Science 310, 1031–1034. 16284181 10.1126/science.1117619

[CIT0082] Walters DR , Avrova A, Bingham IJ, et al. 2012. Control of foliar diseases in barley: towards an integrated approach. European Journal of Plant Pathology 133, 33–73.

[CIT0083] Wei T , Simko V. 2017. R package ‘corrplot’: visualization of a correlation matrix. https://cran.r-project.org/web/packages/corrplot/index.html. Accessed May 2023.

[CIT0084] Yin L , Zhang H, Tang Z, et al. 2021. rMVP: a memory-efficient, visualization-enhanced, and parallel-accelerated tool for genome-wide association study. Genomics, Proteomics & Bioinformatics 19, 619–628. 10.1016/j.gpb.2020.10.007 PMC9040015 33662620

[CIT0085] Zhang C , Dong SS, Xu JY, He WM, Yang TL. 2019. PopLDdecay: a fast and effective tool for linkage disequilibrium decay analysis based on variant call format files. Bioinformatics 35, 1786–1788. 30321304 10.1093/bioinformatics/bty875

[CIT0086] Zhang X , Ovenden B, Milgate A. 2020. Recent insights into barley and *Rhynchosporium commune* interactions. Molecular Plant Pathology 21, 1111–1128. 32537933 10.1111/mpp.12945 PMC7368125

[CIT0087] Zhou X , Stephens M. 2012. Genome-wide efficient mixed-model analysis for association studies. Nature Genetics 44, 821–824. 22706312 10.1038/ng.2310 PMC3386377

