# Supplementary material for: Capitalizing on genebank core collections for rare and novel disease resistance loci to enhance barley resilience
Source: J Exp Bot. 2024 Jun 27;75(18):5940–54. doi: 10.1093/jxb/erae283 (PMC11427843; doi:10.1093/jxb/erae283)
Supplement: erae283_suppl_Supplementary_Figures_S1-S13 [file erae283_suppl_supplementary_figures_s1-s13.pdf]

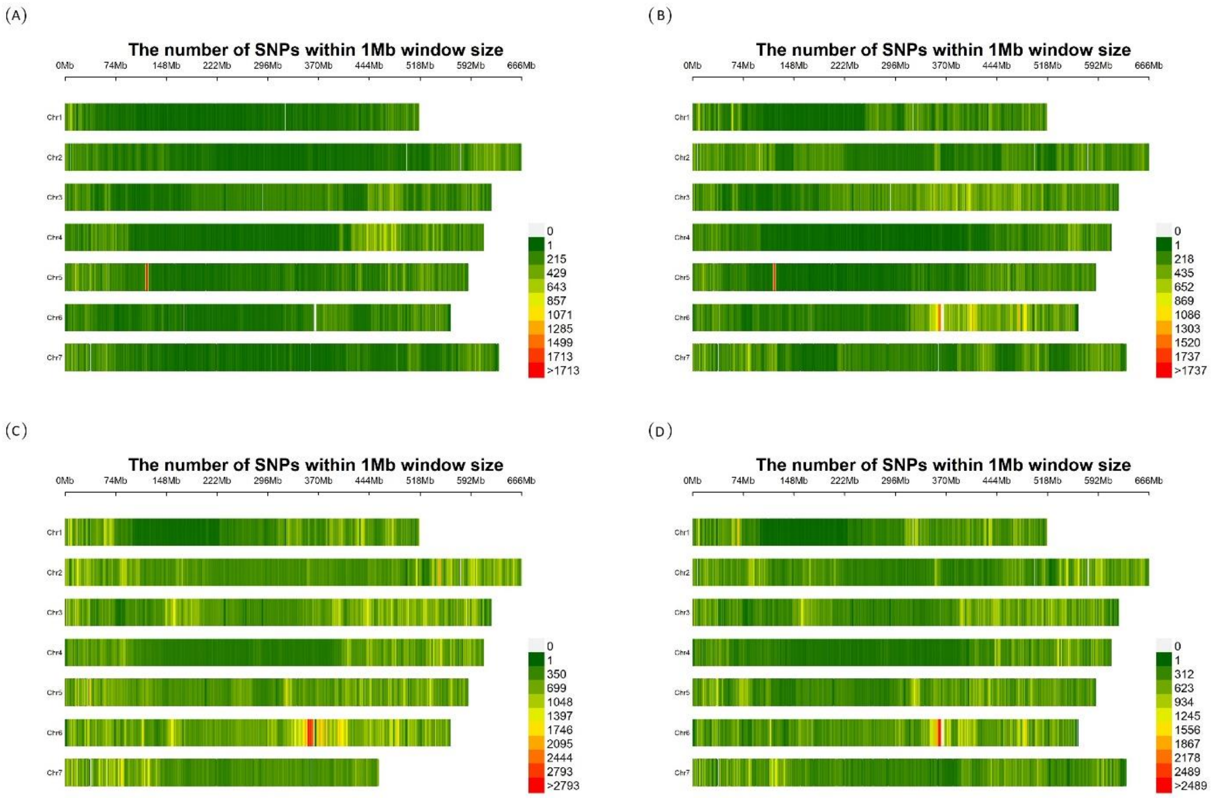

Supplementary Fig. S1 The marker distribution and density of elite line in spring type (A), elite line in winter type (B), plant genetic resources in spring population (C), and plant genetic resources in winter population (D).

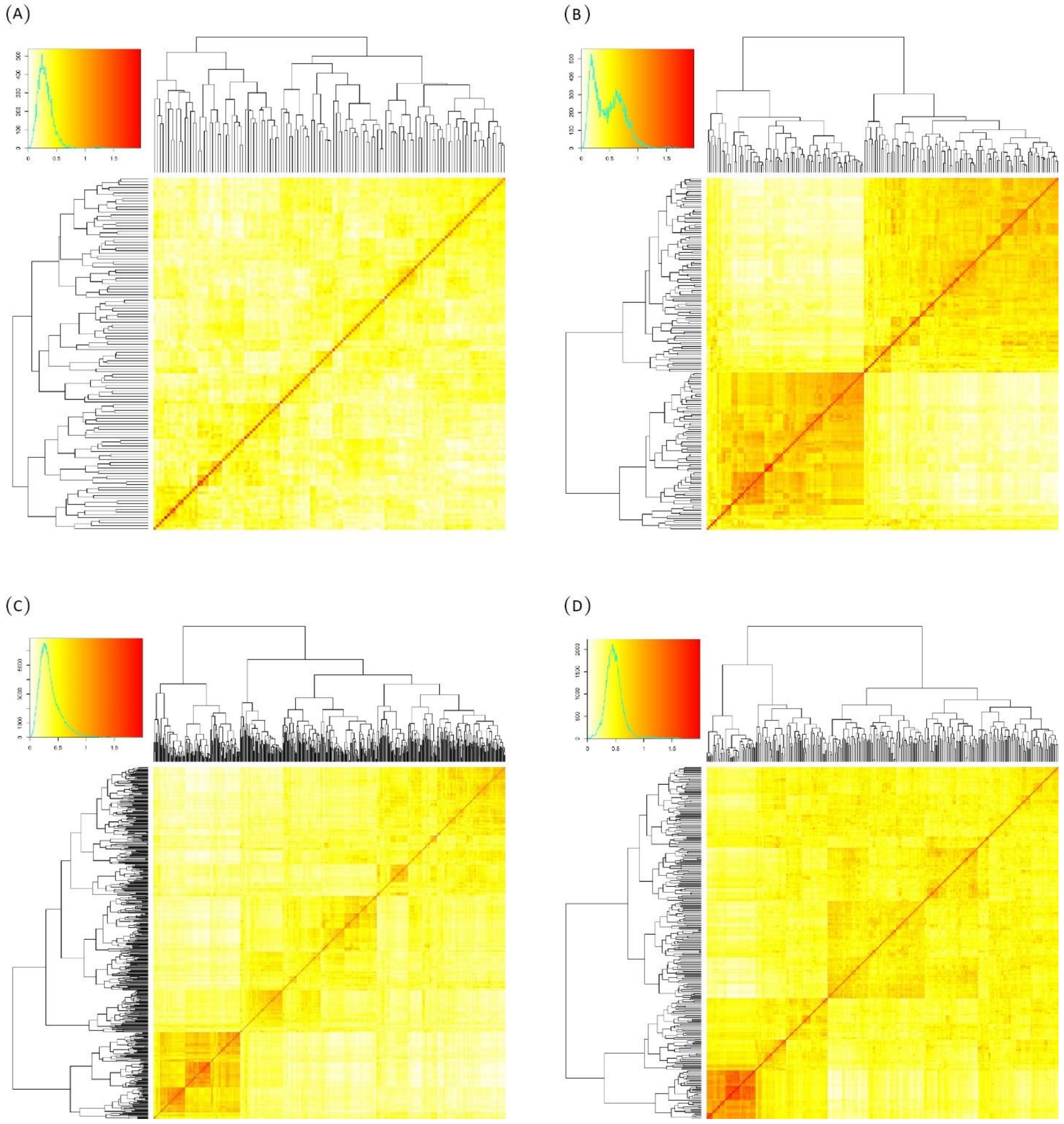

Supplementary Fig. S2 Heatmap of pairwise kinship matrix within elite line in spring type (A), elite line in winter type (B), plant genetic resources in spring type (C), plant genetic resources in winter type (D). A hierarchical clustering tree among individuals was displayed for each sub-group along the top and left axes based on their kinship matrix. Top left, is the distribution of estimated kinship values (the turquoise curve).

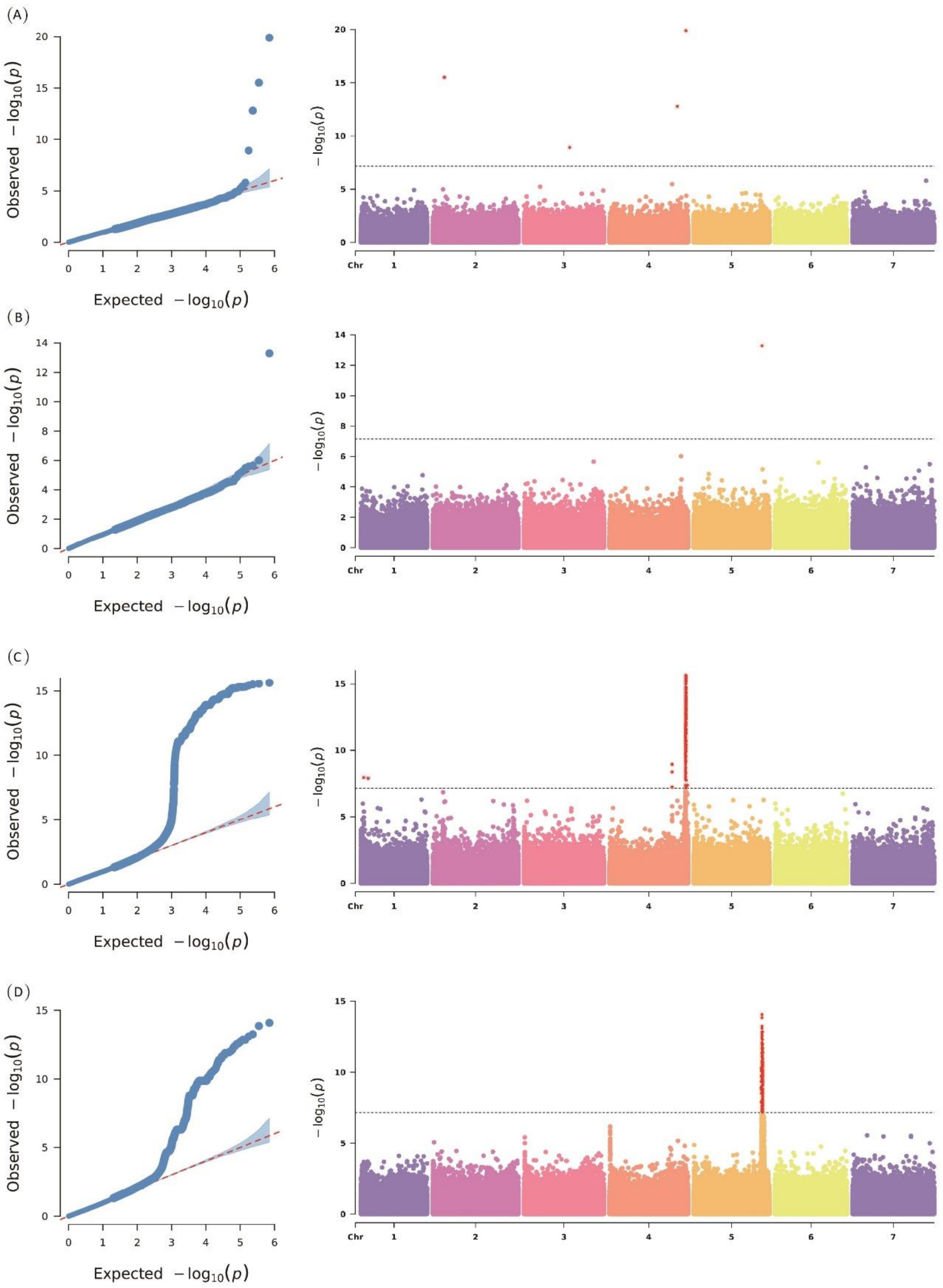

Supplementary Fig. S3 Quantile–quantile plot (left side) and Manhattan plots (right side) of the genome association analysis results in elite line in spring type of the resistance of *Blumeria graminis hordei* (A), *Puccinia hordei* (B) by BLINK, and the resistance of *Blumeria graminis hordei* (C), *Puccinia hordei* (D) by linear mixed model. The black horizontal dashed line in Manhattan plots corresponds to the significance threshold of 7.15.

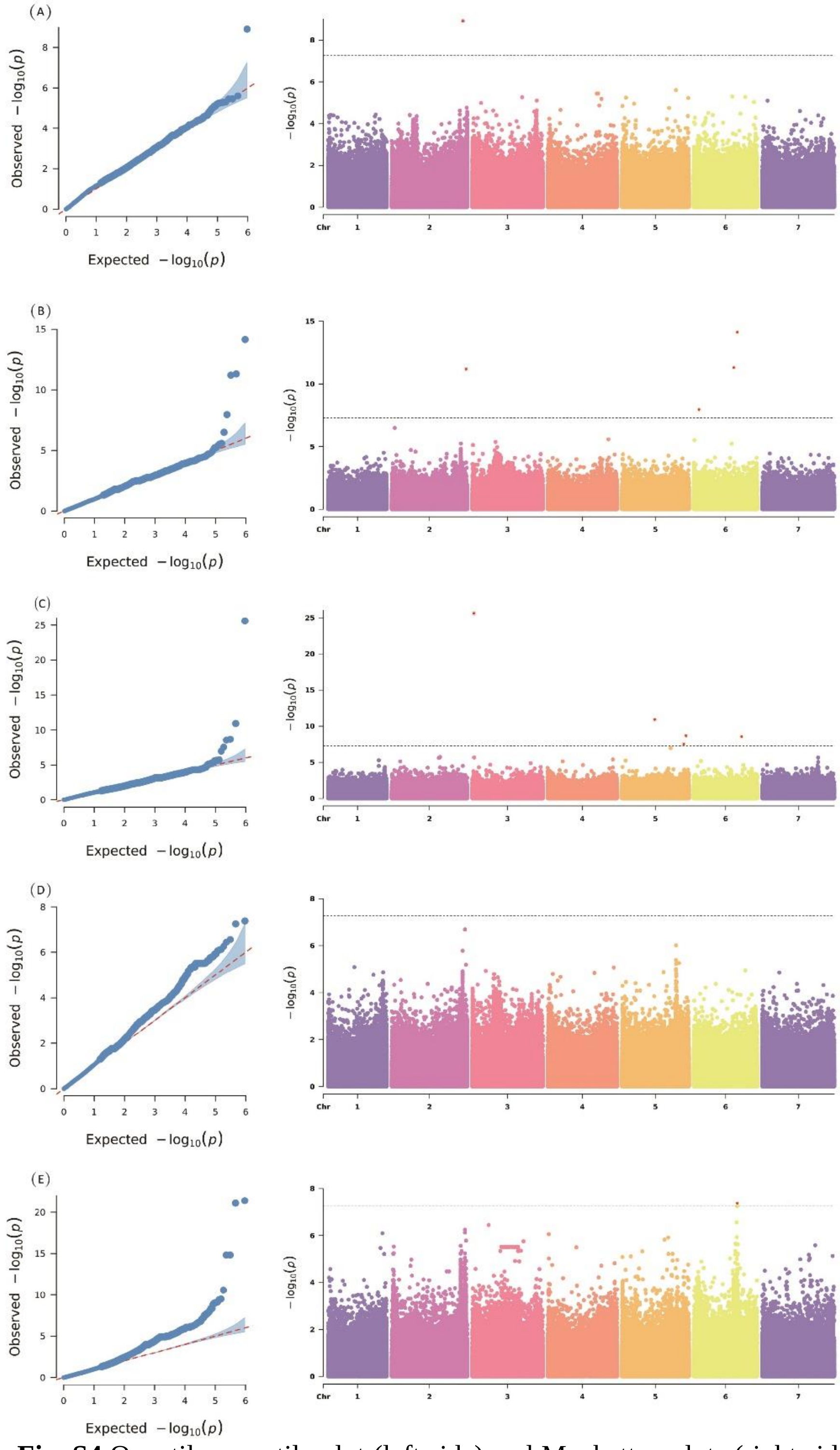

Supplementary Fig. S4 Quantile–quantile plot (left side) and Manhattan plots (right side) of the genome association analysis results in elite line in winter type of the resistance of *Blumeria graminis hordei* (A), *Puccinia hordei* (B), *Rhynchosporium commune* (C) by BLINK, and the resistance of *Blumeria graminis hordei* (D), *Puccinia hordei* (E) by linear mixed model. The black horizontal dashed line in Manhattan plots corresponds to the significance threshold of 7.28.

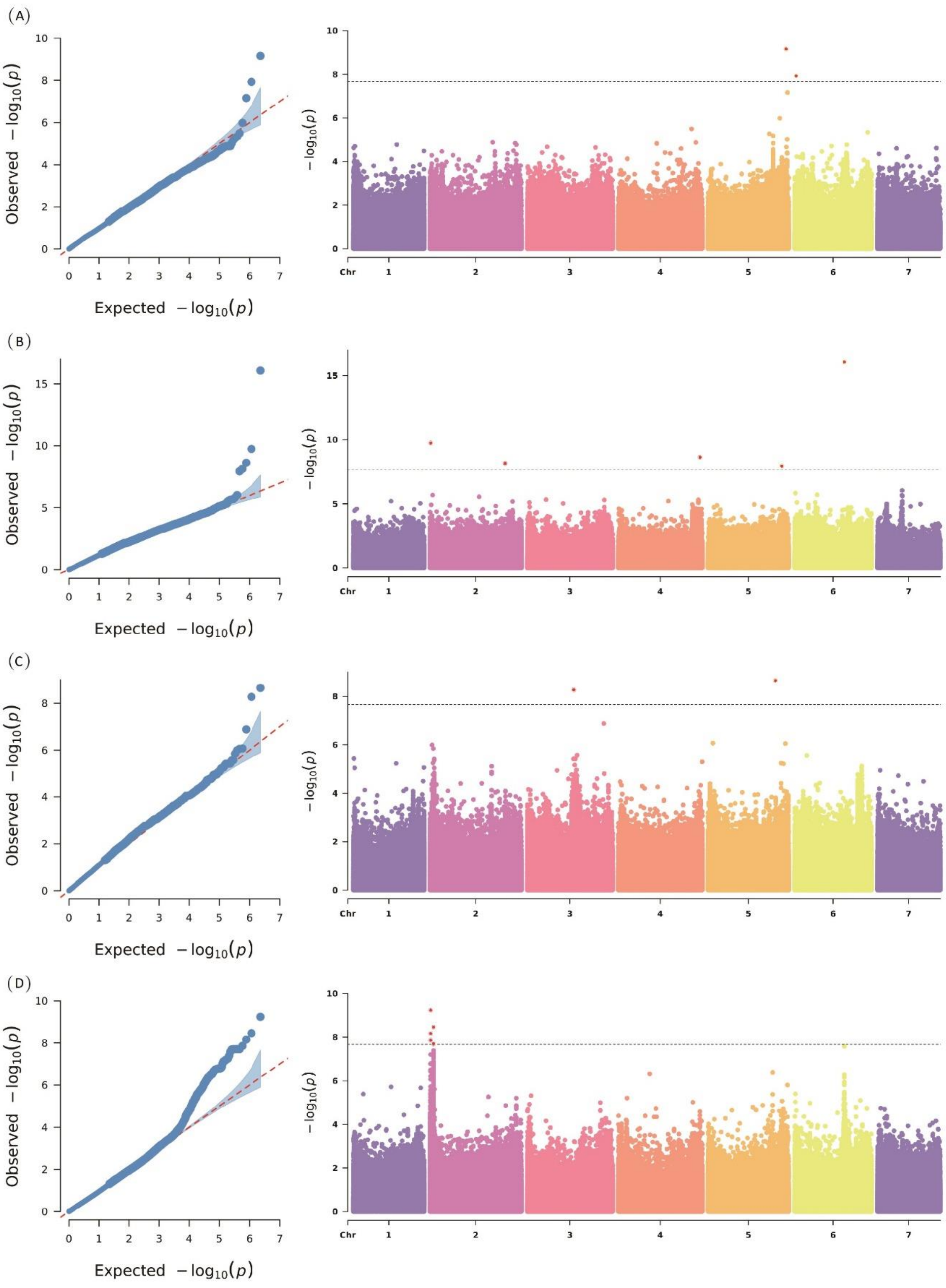

Supplementary Fig. S5 Quantile–quantile plot (left side) and Manhattan plots (right side) of the genome association analysis results in plant genetic resources in spring type of the resistance of *Blumeria graminis hordei* (A), *Puccinia hordei* (B), *Ramularia collo-cygni* (C) by BLINK, and the resistance of *Puccinia hordei* (D) by linear mixed model. The black horizontal dashed line in Manhattan plots corresponds to the significance threshold of 7.67.

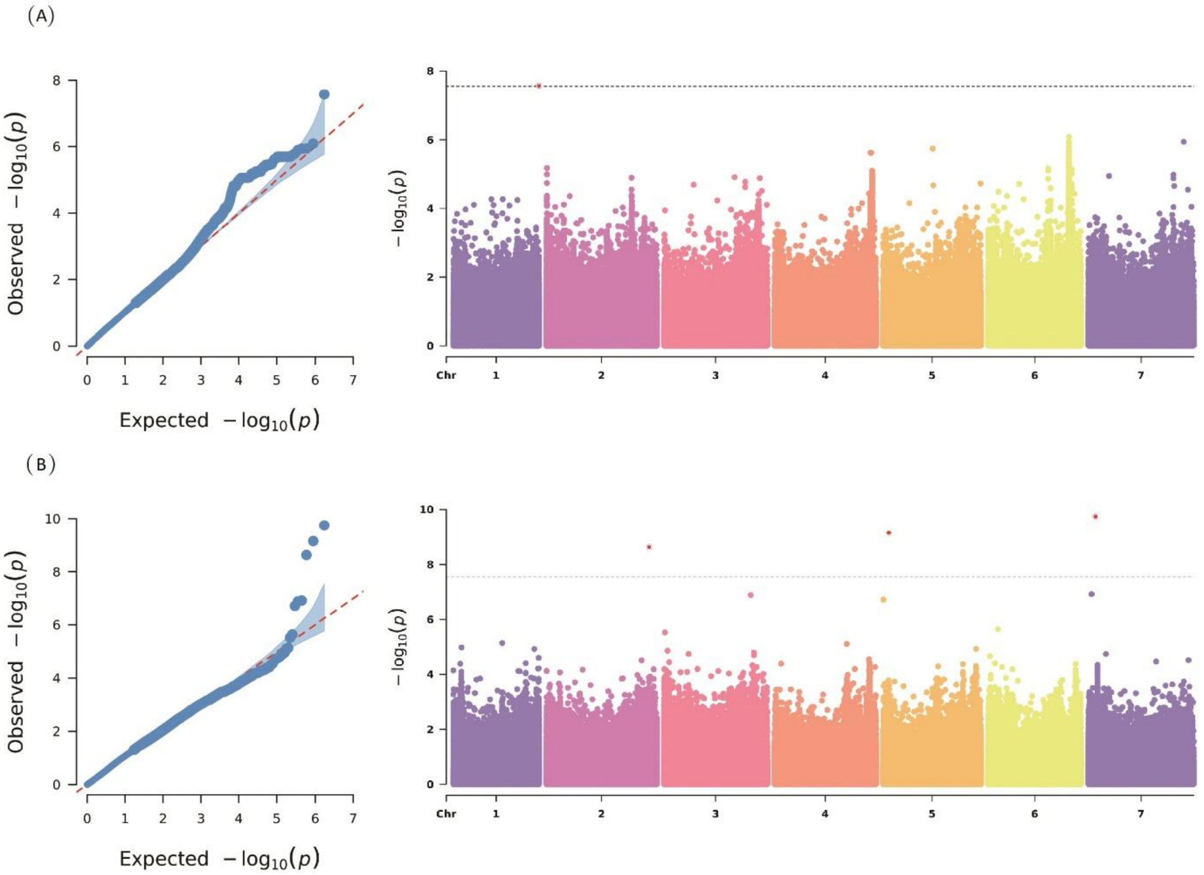

Supplementary Fig. S6 Quantile–quantile plot (left side) and Manhattan plots (right side) of the genome association analysis results in plant genetic resources in winter type of the resistance of *Puccinia hordei* (A), and *Rhynchosporium commune* (B) by BLINK. The black horizontal dashed line in Manhattan plots corresponds to the significance threshold of 7.55.

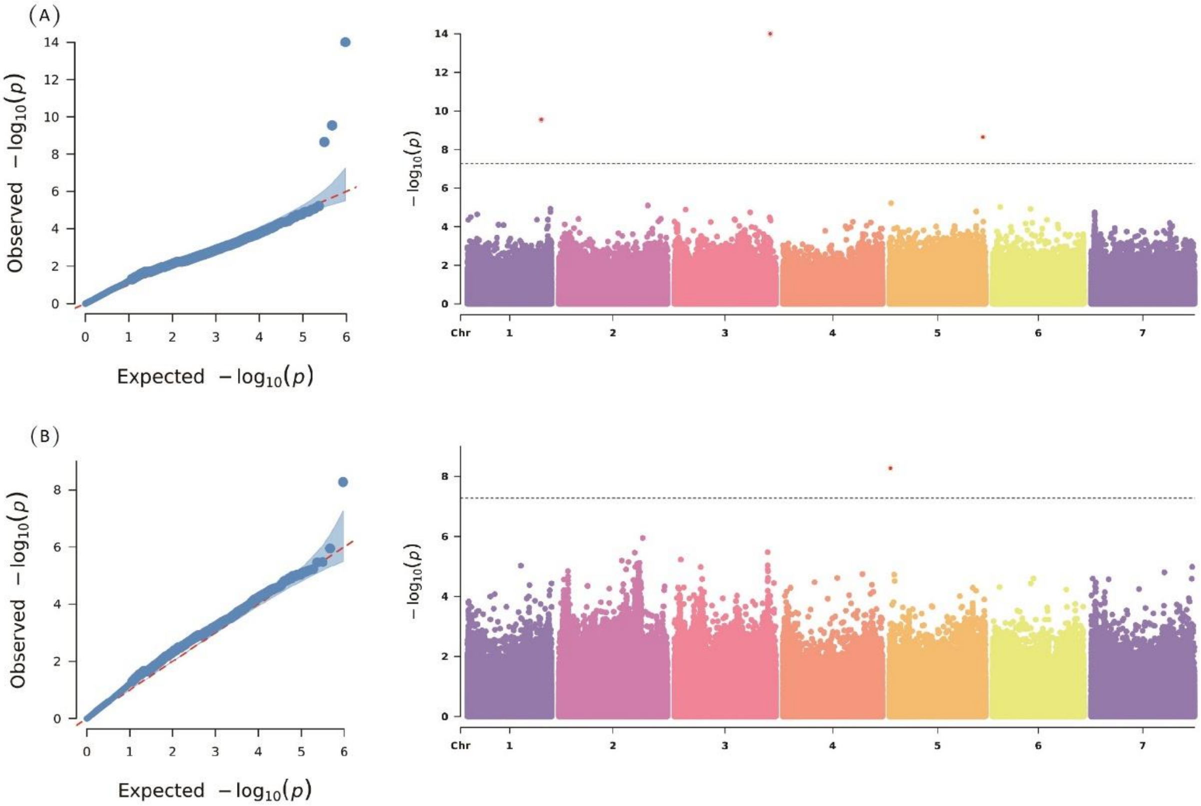

Supplementary Fig. S7 Quantile–quantile plot (left side) and Manhattan plots (right side) of the genome association analysis results in elite line in winter type of heading date (A), and plant height (B) by BLINK. The black horizontal dashed line in Manhattan plots corresponds to the significance threshold of 7.28.

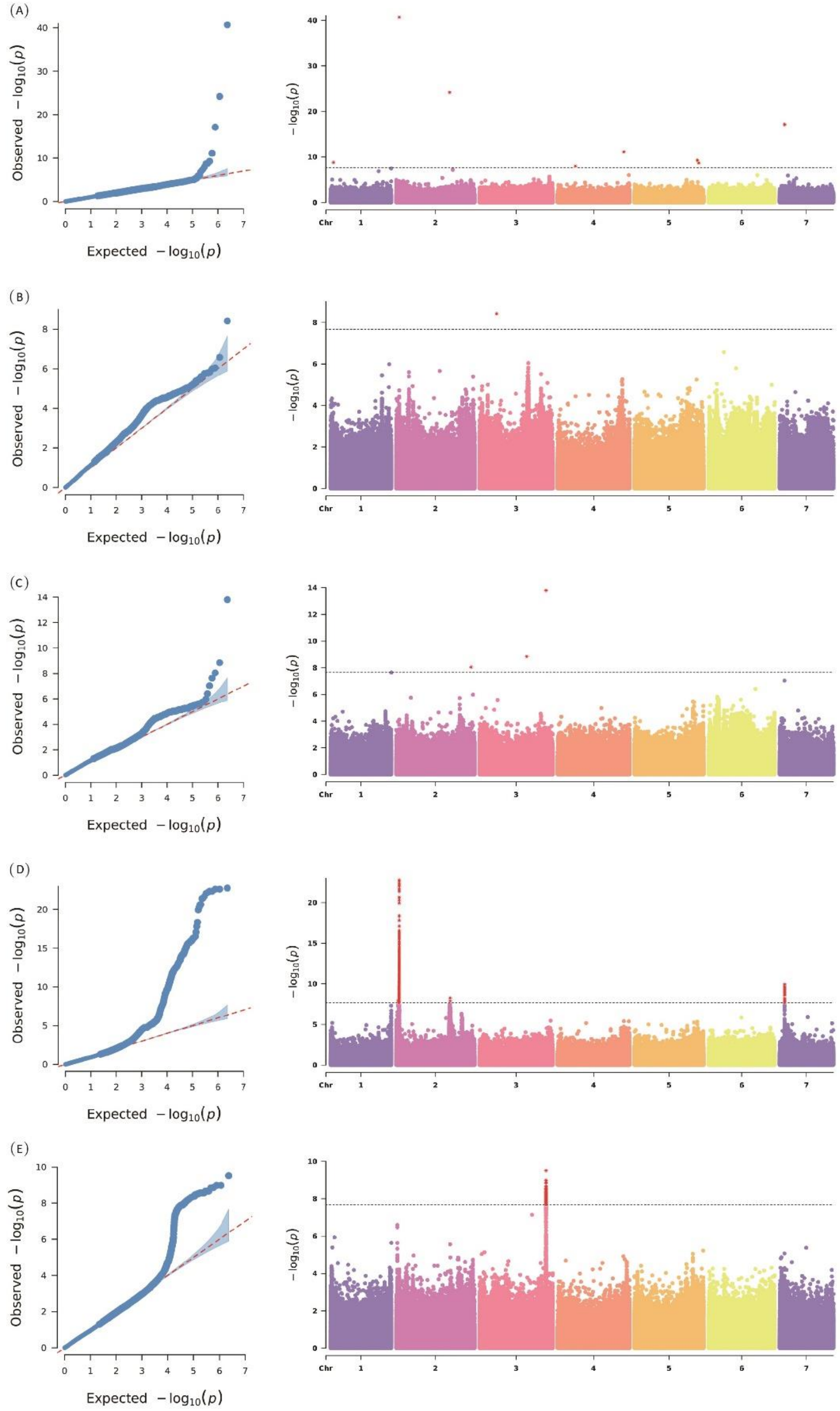

Supplementary Fig. S8 Quantile–quantile plot (left side) and Manhattan plots (right side) of the genome association analysis results in plant genetic resources in spring type of heading date (A), lodging (B), plant height (C) by BLINK, and heading date (D) and plant height (E) by linear mixed model. The black horizontal dashed line in Manhattan plots corresponds to the significance threshold of 7.67.

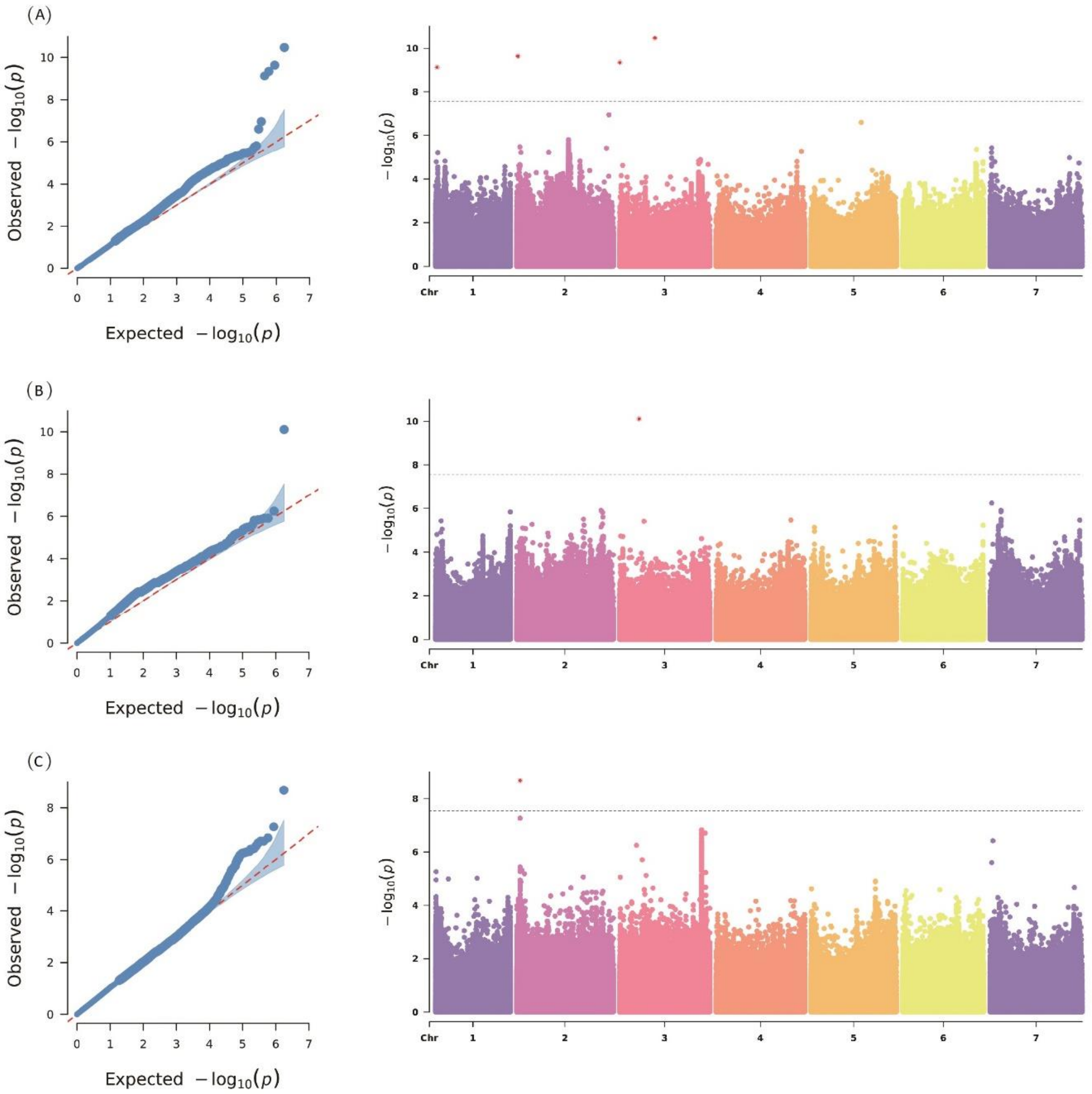

Supplementary Fig. S9 Quantile–quantile plot (left side) and Manhattan plots (right side) of the genome association analysis results in plant genetic resources in winter type of heading date (A), and plant height (B) by BLINK, plant height (C) by linear mixed model. The black horizontal dashed line in Manhattan plots corresponds to the significance threshold of 7.55.

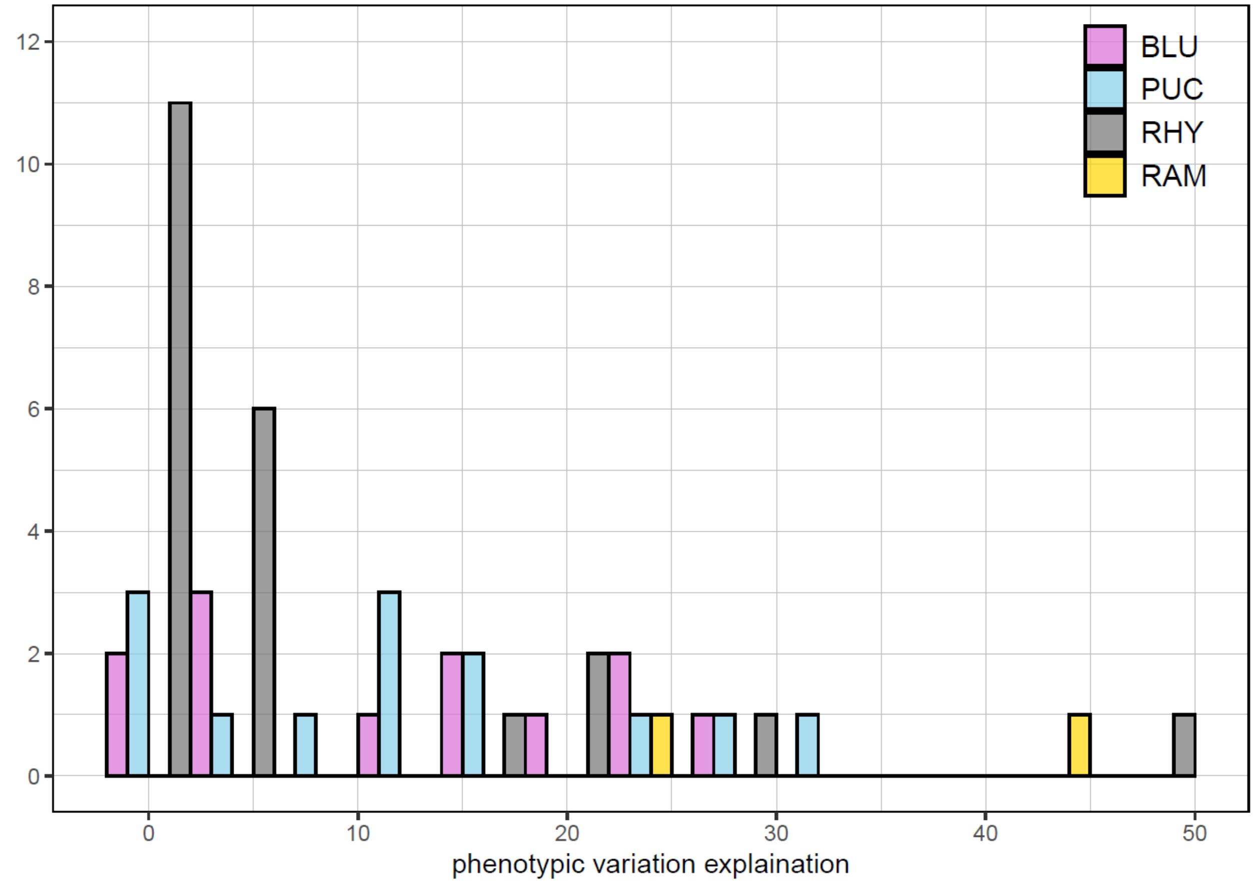

Supplementary Fig. S10 Histogram of the phenotypic variation explained by QTL associated with four diseases traits. BLU: *Blumeria graminis hordei*; PUC: *Puccinia hordei*; RHY: *Rhynchosporium commune*; RAM: *Ramularia collo-cygni*.

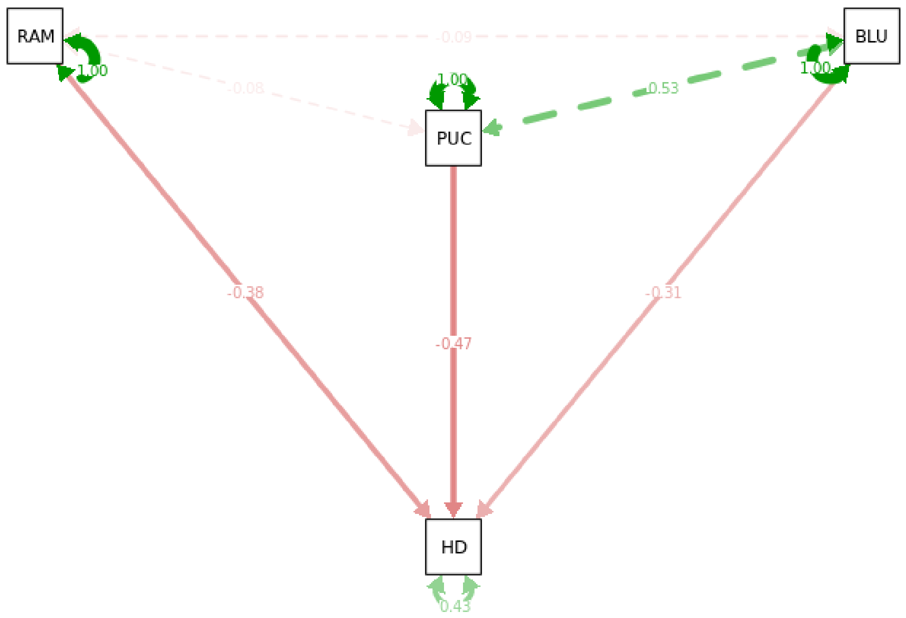

Supplementary Fig. S11 Path analysis between heading date (HD) and other disease resistance traits in spring barley. BLU: *Blumeria graminis hordei*; PUC: *Puccinia hordei*; RAM: *Ramularia collo-cygni*.

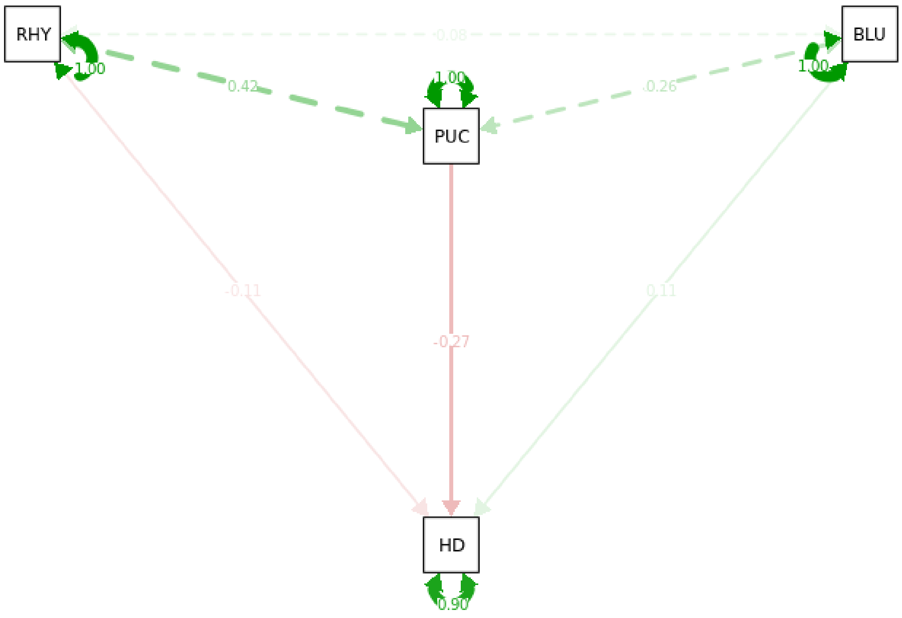

Supplementary Fig. S12 Path analysis between heading date (HD) and other disease resistance traits in winter barley. BLU: *Blumeria graminis hordei*; PUC: *Puccinia hordei*; RHY: *Rhynchosporium commune*.

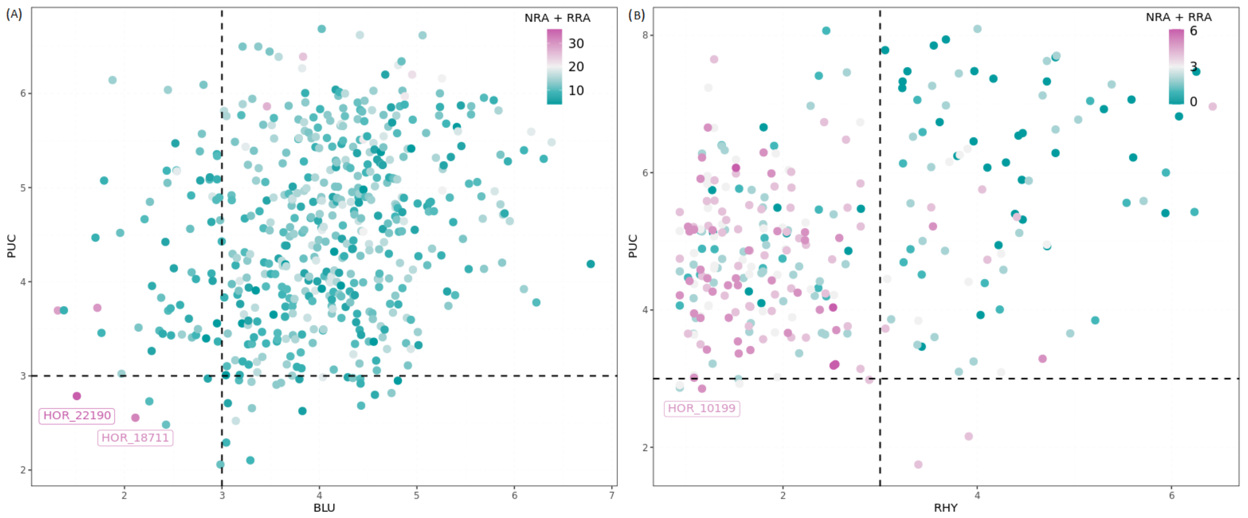

Supplementary Fig. S13 (A) Phenotype distribution of BLU (*Blumeria graminis hordei*) and PUC (*Puccinia hordei*) with the accumulation of the total number of rare resistance allele (RRA) and novel resistance allele (NRA) of plant genetic resources in spring type. (B) Phenotypic distribution of RHY (*Rhynchosporium commune*) and PUC with the accumulation of the total number of RRA and NRA of plant genetic resources in winter type. The color of the point corresponds to the total number of RRA and NRA each genotype harbored.
